# Progressive migration and anagenesis in *Drimys confertifolia* of the Juan Fernández Archipelago, Chile

**DOI:** 10.1007/s10265-014-0666-7

**Published:** 2014-10-08

**Authors:** Patricio López-Sepúlveda, Koji Takayama, Josef Greimler, Daniel J. Crawford, Patricio Peñailillo, Marcelo Baeza, Eduardo Ruiz, Gudrun Kohl, Karin Tremetsberger, Alejandro Gatica, Luis Letelier, Patricio Novoa, Johannes Novak, Tod F. Stuessy

**Affiliations:** 1 Departamento de Botánica, Universidad de Concepción, Casilla 160-C, Concepción, Chile; 2 The University Museum, The University of Tokyo, Hongo 7-3-1, Bunkyo-ku, Tokyo, 113-0033 Japan; 3 Department of Systematic and Evolutionary Botany, Biodiversity Center, University of Vienna, Rennweg 14, 1030 Vienna, Austria; 4 Department of Ecology and Evolutionary Biology and the Biodiversity Institute, University of Kansas, Lawrence, KS 60045, USA; 5 Instituto de Biología Vegetal y Biotecnología, Universidad de Talca, 2 Norte 685, Talca, Chile; 6 Department of Integrative Biology and Biodiversity Research, Institute of Botany, University of Natural Resources and Life Sciences, Gregor Mendel Straße 33, 1180 Vienna, Austria; 7 Laboratorio de Ecofisiología Vegetal, Departamento de Biología, Facultad de Ciencias, Universidad de La Serena, Casilla 599, La Serena, Chile; 8 Centro de Investigaciones en Ecosistemas, Universidad Nacional Autónoma de México, C.P. 58190 Morelia, Michoacán Mexico; 9 Jardín Botánico de Viña del Mar, Corporación Nacional Forestal, Camino El Olivar 305, Viña del Mar, Chile; 10 Institute for Applied Botany and Pharmacognosy, University of Veterinary Medicine, Veterinärplatz 1, 1210 Vienna, Austria; 11 Herbarium, Department of Evolution, Ecology, and Organismal Biology, The Ohio State University, 1315 Kinnear Road, Columbus, OH 43212, USA

**Keywords:** AFLPs, Anagenesis, Genetic variation, Microsatellites, Oceanic islands, Migration

## Abstract

A common mode of speciation in oceanic islands is by anagenesis, wherein an immigrant arrives and through time transforms by mutation, recombination, and drift into a morphologically and genetically distinct species, with the new species accumulating a high level of genetic diversity. We investigate speciation in *Drimys confertifolia*, endemic to the two major islands of the Juan Fernández Archipelago, Chile, to determine genetic consequences of anagenesis, to examine relationships among populations of *D. confertifolia* and the continental species *D. winteri* and *D. andina*, and to test probable migration routes between the major islands. Population genetic analyses were conducted using AFLPs and nuclear microsatellites of 421 individuals from 42 populations from the Juan Fernández islands and the continent. *Drimys confertifolia* shows a wide genetic variation within populations on both islands, and values of genetic diversity within populations are similar to those found within populations of the continental progenitor. The genetic results are compatible with the hypothesis of high levels of genetic variation accumulating within anagenetically derived species in oceanic islands, and with the concept of little or no geographical partitioning of this variation over the landscape. Analysis of the probability of migration within the archipelago confirms colonization from the older island, Robinson Crusoe, to the younger island Alejandro Selkirk.

**Electronic supplementary material:**

The online version of this article (doi:10.1007/s10265-014-0666-7) contains supplementary material, which is available to authorized users.

## Introduction

Patterns and processes of speciation in oceanic islands have long captured the attention of evolutionary biologists (Drake et al. 2002; Rosindell and Phillimore 2011; Schaefer et al. 2011; Stuessy and Ono 1998). Important attributes of oceanic islands, such as geographical isolation, clearly delimited area, and restricted fauna and flora, have led to islands being regarded as natural laboratories for the study of evolution. They offer countless opportunities for investigating evolutionary processes in detail, especially for studying genetic, ecological, biogeographic, reproductive, and morphological divergence (Moore et al. 2002; Mort et al. 2002).

Numerous hypotheses and discussions regarding processes of speciation in oceanic islands have occurred (Carlquist 1974; Grant et al. 1996; Stuessy et al. 2006). The most commonly described speciation mechanism in islands is through cladogenesis. In this process, after a single introduction, numerous lineages diverge rapidly from the founding population as they adapt to different habitats with appropriate adaptations (Schluter 2000). The genetic consequence of this process is low level of genetic variation within and among populations of each species (Baldwin et al. 1998; Crawford and Stuessy 1997; Emerson 2002; Johnson et al. 2000; Stuessy et al. 2006). Examples of this mechanism of divergence and speciation in oceanic islands are numerous, such as *Aeonium* (Crassulaceae) and *Echium* (Boraginaceae) in the Canary Islands (Böhle et al. 1996; Jorgensen and Olesen 2001), *Dendroseris* and *Robinsonia* (Asteraceae) in the Juan Fernández Archipelago (Crawford et al. 1998), *Bidens* (Asteraceae), *Schiedea* (Caryophyllaceae), *Cyanea*, *Lobelia* and *Trematolobelia* (Lobeliaceae) in the Hawaiian Islands (Givnish et al. 2009; Knope et al. 2012; Price and Wagner 2004), and *Scalesia* (Asteraceae) in the Galápagos Islands (Eliasson 1974; Schilling et al. 1994).

The other major type of speciation in oceanic islands is anagenesis, also called simple geographic or phyletic speciation (Simpson 1953). In this case, after colonizers establish a population on a new island, the processes of drift, recombination, and mutation modify the composition of the original pioneer population and over time genetic variation accumulates by the processes of recombination, and mutation. The final result is a new species that differs genetically and morphologically from its ancestor, with levels of genetic variation approximating those of the progenitor species (Stuessy et al. 2006). Examples of this type of speciation are less frequent, but it has been documented in *Dystaenia* (Apiaceae; Pfosser et al. 2006) and *Acer* (Sapindaceae; Takayama et al. 2012a, b) of Ullung Island, Korea, and in *Weigela* (Caprifoliaceae; Yamada and Maki 2012) of the Izu Islands, Japan. The genetic consequences of anagenetic speciation show relatively high levels of genetic differentiation within and among populations of the island endemic relative to continental source populations. Obviously, many factors impact levels of genetic variation within island populations, such as breeding systems, island age, human impact, etc. (Stuessy et al. 2013), but mode of speciation is particularly significant.

Also important for understanding patterns and processes of evolution in oceanic islands is inferring routes of migration among islands within archipelagos. The classical hypothesis regarding archipelagos has assumed a single colonization event from a continental area first to the oldest island and subsequent colonization of the younger islands (Juan et al. 2000; Gillespie and Roderick 2002), the so-called “progression rule” (Funk and Wagner 1995). Although this concept relies on parsimony, which is not unreasonable, other possibilities have been demonstrated, such as reverse colonization (Ballemain and Ricklefs 2008; Carine et al. 2004), colonization followed by extinction, or migration from younger to older islands (Emerson 2002; Juan et al. 2000). Obviously important also are availability of transportation vectors (Gillespie and Roderick 2002) and adaptation and dispersal of propagules (Cowie and Holland 2006).

**Fig. 1:**
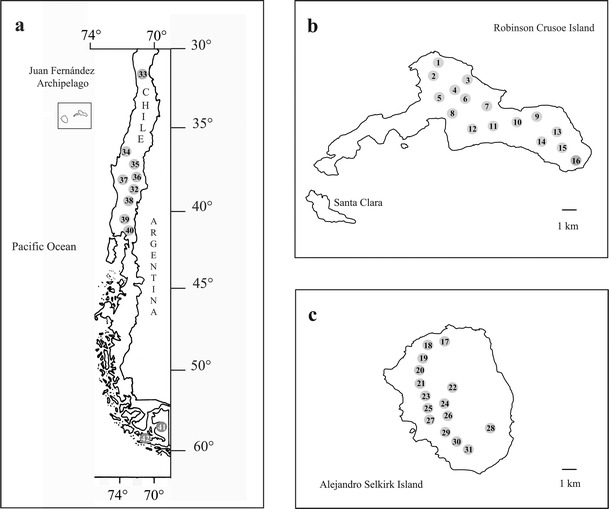
Geographical position of populations sampled of *Drimys winteri* and *D.* *andina* in continental Chile (**a**) and *Drimys confertifolia* in Robinson Crusoe (**b**) and Alejandro Selkirk (**c**) Islands

An appropriate group of islands in which to study anagenetic speciation and migration is the Juan Fernández Archipelago, located in the Pacific Ocean 667 km W of continental Chile (33°S/78–80°W, Fig. 1). The archipelago consists of two main islands, Robinson Crusoe (=Masatierra) and Alejandro Selkirk (=Masafuera), separated by 181 kms. At present the islands are approximately of equal size (50 km^2^, Stuessy 1995), but they differ in geological age, c. 4 million years old for Robinson Crusoe Island and 1–2 million years old for Alejandro Selkirk Island (Stuessy et al. 1984). The native vascular flora of the archipelago includes 75 families, 213 genera, and 361 species, with a 12 % endemism at the generic level, and 60 % at the specific level (Marticorena et al. 1998).

A suitable genus to study the genetic consequences of anagenetic speciation and migration in the Juan Fernández Archipelago is *Drimys* (Winteraceae). The genus contains seven species distributed in Central and South America (Ehrendorfer et al. 1979; Marquínez et al. 2009; Rodríguez and Quezada 2001; Smith 1943), two of which, *D. andina* and *D. winteri* (with two varieties, var. *winteri* and var*. chilensis*), grow in continental Chile, with the others disjunct in Brazil and northwestern South America (with extensions northward into Mexico). *Drimys* *confertifolia* is endemic to the Juan Fernández Archipelago, common on Robinson Crusoe Island, and with a patchy distribution on Alejandro Selkirk Island. These three species comprise a closely related complex, as evidenced by very similar ITS sequences (Ruiz et al. 2008).

In recent years the number of molecular markers available to study genetic diversity and phylogeny in islands has increased significantly (Emerson 2002). Amplified Fragment Length Polymorphisms (AFLPs, Vos et al. 1995), a dominant marker, has been shown to provide a good overall measure of genetic diversity and structure at the population level (Tremetsberger et al. 2003). Nuclear microsatellites are co-dominant highly polymorphic markers that are widely used to study the genetic structure of populations and migration routes (Hardy et al. 2006). In this study, therefore, we selected both methodologies to analyze the genetic consequences of anagenesis and migration in *Drimys confertifolia*.

The objectives of this paper are to: (1) determine genetic relationships and variation within and among populations of *D. confertifolia*, *D. andina*, and *D. winteri*; (2) establish the most probable migration route(s) of the endemic species between islands; and (3) assess genetic consequences of island anagenetic speciation.

## Materials and methods

### Species

**Fig. 2:**
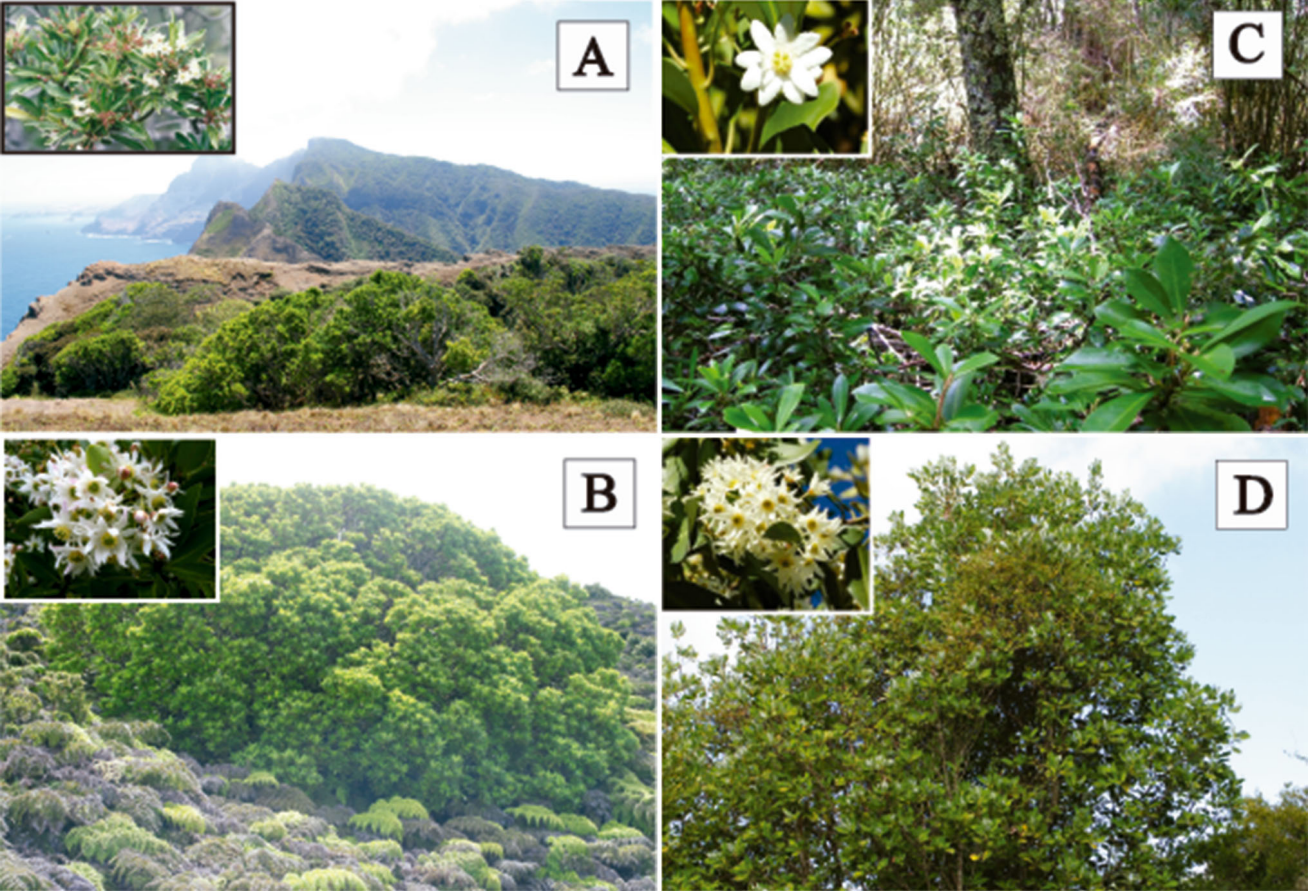
Habitats and flowers (*insets*) of *D. confertifolia* on Robinson Crusoe Island (**a**), *D. confertifolia* on Alejandro Selkirk Island (**b**), *D. andina* (**c**), and *Drimys winteri* var. *winteri* (**d**)

*Drimys confertifolia* Phil., “Canelo” (Fig. 2a, b), is a protogynous (and therefore out-crossing) tree (Bernardello et al. 2001) to 15 m tall, endemic to the Juan Fernández Archipelago. Chromosomally the species is known as *n* = c. 43 (Sun et al. 1990), which is probably at the dodecaploid level (based on *x* = 7, Raven and Kyhos 1965). This is the same level reported for *D. winteri* (Raven and Kyhos 1965) and *D. granadensis* (Ehrendorfer et al. 1979). In Robinson Crusoe Island *D. confertifolia* is common, growing together with *Myrceugenia* *fernandeziana* (Myrtaceae), *Fagara mayu* (Rutaceae), and *Juania australis* (Arecaceae) (Greimler et al. 2002). In Alejandro Selkirk Island it occurs in patches or is scattered, not forming large pure stands, and growing together with the ferns *Blechnum cycadifolium* (Blechnaceae), *Dicksonia externa* (Dicksoniaceae), *Histiopteris incisa* (Dennstaedtiaceae), and *Lophosoria quadripinnata* (Dicksoniaceae) (Greimler et al. 2013). *Drimys confertifolia* is hermaphroditic and wind-pollinated (Bernardello et al. 2001), flowering from November to January (Rodríguez and Quezada 2001).

The continental species included in this study are *D*. *andina* (Reiche) R.A.Rodr. et Quez. This species (“Canelo Enano” Fig. 2c), grows as a small shrub to 1.5 m tall, and is endemic to the subantarctic forest distributed along the Andes and occasionally in the Coastal Cordillera (37°–41° S) (Rodríguez and Quezada 2001). The other species is *D*. *winteri* J.R.Forst. et G.Forst. (Fig. 2d) with two recognized varieties, var. *winteri* (“Canelo, foye”) and var. *chilensis* (DC.) A.Gray (“Canelo”). The former variety is a tree to 17 m, and is found in the subantarctic forests in the extreme southern portion of Chile (45°44′–55°58′ S) (Rodríguez and Quezada 2001). The latter variety is a large tree (to 20 m) and is common and endemic to continental Chile, growing from 0 to 1,700 m in the Coastal Cordillera and the Andes Mountains between 30°20′–46°25′ S (Rodríguez and Quezada 2001).

### Collection and DNA isolation

The species were collected during expeditions to the Juan Fernández Archipelago in 2010 and 2011. Leaves of *D*. *confertifolia* were collected in silica gel from individuals of 16 populations on Robinson Crusoe Island (Nos. 1–16) and from 15 populations on Alejandro Selkirk Island (Nos. 17–31) (Fig. 1). In continental Chile, samples came from one population of *D*. *andina* (No. 32), two populations of *D*. *winteri* var. *winteri* (Nos. 41 and 42), and eight populations of *D*. *winteri* var. *chilensis* (Nos. 33–40) were collected (Fig. 1; Table 1). Voucher specimens of each population are deposited in the herbarium of the University of Vienna (WU). The DNeasy 96 Plant Kit (Qiagen, Hilden, Germany) was used for extraction of DNA for AFLP and microsatellite analyses. Details of numbers of individuals and populations used for AFLP and microsatellites, and their distributions, are given in Table 1 and Fig. 1.

**Table 1:** Values of genetic diversity and divergence based on AFLP and nuclear microsatellite analysis in 42 populations of *Drimys confertifolia*, *D. andina*, *D*. *winteri* var. *winteri* and *D*. *winteri* var. *chilensis*

AFLPs									Microsatellites								
Taxon and vouchers	*N*	N°	PPB	TNB	SDI	AGDOL	RI	NPB	N°	*H*_O_	*uH*_E_	*F*_IS_	*N*_A_	*A*_R_	*H*_E_	*N*_PA_	*N*_LCA_
*D. confertifolia* Robinson Crusoe Island																	
19130	1	11	68.4	442	106.84	0.26	1.64	0	10	0.44	0.66	0.29*	4.50	4.73	0.63	0.00	0.75
19134	2	12	70.7	456	109.33	0.25	1.71	2	12	0.48	0.67	0.25*	4.88	4.23	0.64	0.00	0.50
19185	3	12	66.6	441	105.65	0.25	1.56	0	12	0.50	0.64	0.21	4.88	4.35	0.61	0.00	0.50
19173	4	10	66.9	448	108.55	0.26	1.79	1	10	0.46	0.68	0.29*	5.25	4.79	0.65	0.13	0.63
19209	5	14	67.6	431	99.91	0.23	1.44	0	15	0.52	0.66	0.19*	5.00	4.16	0.64	0.00	0.75
19108	6	11	71.7	453	114.60	0.27	1.59	0	11	0.51	0.67	0.24	5.50	4.30	0.64	0.13	0.88
19191	7	10	70.3	445	112.58	0.28	1.78	1	10	0.45	0.64	0.28*	4.75	4.25	0.61	0.00	0.63
19290	8	4	42.4	319	76.58	0.23	1.27	0	3	0.54	0.68	0.00	3.13	–	0.57	0.00	0.63
19284	9	9	60.0	404	96.89	0.23	1.34	0	9	0.46	0.52	0.02	4.00	4.06	0.49	0.00	0.63
19227	10	12	67.4	426	103.15	0.24	1.41	1	13	0.48	0.68	0.26*	5.38	4.47	0.65	0.00	1.00
19275	11	15	72.9	451	111.59	0.25	1.59	0	15	0.43	0.53	0.15	4.25	3.99	0.52	0.00	0.75
19197	12	8	55.2	390	90.76	0.23	1.36	0	8	0.31	0.66	0.48*	4.38	4.64	0.62	0.00	0.63
19268	13	14	67.9	430	102.99	0.24	1.35	0	13	0.49	0.62	0.13	4.63	4.52	0.60	0.00	0.50
19256	14	14	65.2	435	100.53	0.23	1.41	0	14	0.47	0.68	0.26*	5.50	4.04	0.66	0.25	0.63
19260	15	14	66.9	434	102.29	0.23	1.37	0	14	0.54	0.67	0.13	5.50	3.45	0.65	0.13	1.50
19248	16	14	63.6	416	94.05	0.22	1.38	1	15	0.60	0.66	0.04	4.88	3.61	0.64	0.00	1.00
	Average (±SD)		66.8 (±4.5)	433.5 (±18.5)	103.98 (±6.88)	0.24 (±0.17)	1.50 (±1.61)	0.40 (±0.63)		0.48 (±0.063)	0.64 (±0.051)	0.21 (±0.11)	4.89 (±0.48)	4.24 (±0.38)	0.62 (±0.049)	0.04 (±0.078)	0.75 (±0.26)
*D. confertifolia* Alejandro Selkirk Island																	
20041	17	9	54.5	398	86.01	0.21	1.69	0	10	0.40	0.51	0.16	4.25	3.25	0.48	0.00	0.50
20014	18	17	73.8	474	108.87	0.25	2.02	0	16	0.31	0.48	0.19*	4.13	3.50	0.46	0.00	0.63
20013	19	1	0.0	220	0.00	0.00	0.00	0	1	0.63	0.63	–1.00	1.63	–	0.31	0.00	0.38
19813	20	2	24.7	302	49.91	0.25	1.60	0	3	0.29	0.33	–0.14	1.88	–	0.27	0.00	0.25
20005	21	3	28.8	328	54.71	0.19	1.88	0	3	0.21	0.49	0.35	2.25	–	0.41	0.00	0.38
20023	22	1	0.0	154	0.00	0.00	0.00	1	1	0.38	0.38	–1.00	1.38	–	0.19	0.00	0.00
19803	23	10	54.2	409	85.89	0.20	1.69	0	10	0.38	0.44	0.14	3.13	3.27	0.42	0.00	0.13
19638	24	1	0.0	258	0.00	0.00	0.00	0	1	0.50	0.50	–1.00	1.50	–	0.25	0.00	0.13
19810	25	3	28.1	327	53.25	0.19	2.06	1	3	0.29	0.43	0.10	2.25	–	0.36	0.00	0.13
20049	26	3	30.9	319	59.49	0.21	1.58	0	3	0.33	0.46	0.11	2.38	–	0.38	0.00	0.00
19616	27	2	23.7	272	47.83	0.24	1.47	0	2	0.50	0.58	–0.14	2.25	–	0.44	0.00	0.00
19652	28	8	57.3	431	90.75	0.23	2.14	0	7	0.41	0.59	0.26	3.25	3.25	0.54	0.00	0.50
19656	29	9	61.2	440	92.61	0.23	2.17	0	12	0.30	0.52	0.29*	4.00	2.89	0.50	0.00	0.50
19657	30	19	65.4	426	96.56	0.21	1.59	1	18	0.36	0.49	0.19*	4.13	3.31	0.48	0.13	0.88
19658	31	8	45.1	325	73.05	0.18	1.05	0	7	0.27	0.44	0.27	3.25	3.79	0.41	0.00	0.38
	Average (±SD)		58.8 (±9.2)	414.7 (±46.4)	90.53 (±10.99)	0.22 (±0.02)	1.76 (±0.39)	0.14 (±0.38)		0.35 (±0.05)	0.50 (±0.05)	0.21 (±0.06)	3.73 (±0.50)	3.3 (±0.27)	0.47 (±0.045)	0.02 (±0.05)	0.50 (±0.23)
*D. andina*																	
4324	32	16	60.4	383	91.92	0.20	1.18	0	13	0.37	0.49	0.17	4.13	3.92	0.48	0.25	1.00
*D. winteri* var. *chilensis*																	
4	33	14	55.6	344	82.73	0.18	1.19	1	13	0.38	0.65	0.41*	4.63	3.83	0.63	0.00	0.75
4314	34	14	37.7	243	60.36	0.14	0.78	0	13	0.44	0.69	0.33*	5.13	3.71	0.66	0.00	1.63
4301	35	12	47.0	301	73.14	0.17	0.99	0	11	0.53	0.62	0.07	4.13	4.24	0.59	0.00	0.75
4300	36	12	47.2	302	70.04	0.16	1.03	1	12	0.51	0.60	0.11	4.38	4.65	0.58	0.00	0.75
2426	37	11	45.8	299	71.45	0.17	1.01	0	11	0.44	0.58	0.14	4.25	4.54	0.56	0.13	1.00
4321	38	15	40.3	264	61.32	0.14	0.76	0	13	0.37	0.69	0.44*	5.63	4.09	0.66	0.25	1.63
4330	39	18	53.0	320	81.65	0.18	0.84	0	18	0.49	0.69	0.27*	6.00	3.75	0.67	0.00	1.75
2401	40	8	58.8	355	101.19	0.24	1.04	1	10	0.49	0.60	0.11	4.63	3.84	0.57	0.13	1.13
*D. winteri* var. *winteri*																	
2402	41	10	41.7	272	64.47	0.16	0.92	0	10	0.58	0.58	–0.05	3.75	3.53	0.55	0.00	0.50
2420	42	12	43.2	303	66.76	0.15	0.97	0	12	0.46	0.55	0.09	4.63	3.44	0.52	0.00	1.00
	Average (±SD)		47.0 (±6.9)	300.3 (±34.5)	73.31 (±12.39)	0.17 (±0.03)	0.95 (±0.13)	0.3 (±0.48)		0.47 (±0.06)	0.62 (±0.05)	0.19 (±0.16)	4.71 (±0.69)	3.96 (±0.41)	0.60 (±0.05)	0.05 (±0.09)	1.09 (±0.44)

Population data in italics, with four or fewer individuals, were not included in the statistical analyses

*N* assigned population number, *N°* number of individuals analyzed, *PPB* percentage of polymorphic bands, *TNB* total number of bands, *SDI* Shannon diversity index, *AGDOL* average gene diversity over loci, *RI* rarity index, *NPB* number of private bands, *H*_O_ observed heterozygosity, *µH*_*E*_ expected heterozygosity, *F*_*IS*_ inbreeding coefficient, *N*_*A*_ number of alleles per locus, *A*_*R*_ allelic richness, *H*_*E*_ expected proportion of heterozygotes, *N*_*PA*_ number of private alleles, *N*_*LCA*_ number of locally common alleles (freq. ≥5 %) found in 25 % or fewer populations, – no data because the number was calculated with populations that contained seven or more individuals, * significant *F*_IS_ values (*P* < 0.05) after Bonferroni correction

### Genetic markers

For AFLPs we followed the protocol of Vos et al. (1995) with modifications by Tremetsberger et al. (2003). For selection of primers, a trial was done with 85 primer combinations and four individuals from each of six populations representative of all taxa and islands. The following five primer combinations were selected: *Mse*I–CTG/*Eco*RI–ACA (FAM); *Mse*I–CTC/*Eco*RI–ACA (FAM); *Mse*I–CAC/*Eco*RI–ATG (VIC); *Mse*I–CAG/*Eco*RI–AAG (VIC); and *Mse*I–CTC/*Eco*RI–AGC (NED). A total of 421 individuals was analyzed, 279 from *D*. *confertifolia*, 16 from *D*. *andina*, 104 from *D*. *winteri* var. *chilensis*, and 22 from *D*. *winteri* var. *winteri* (for details see Table 1). Amplified fragments of DNA were run on an automated sequencer (ABI 3130xl, Applied Biosystems, CA, USA).

Fragments were scored using the program GeneMarker ver. 1.85 (SoftGenetics LLC, PA, USA). The range for allele call was 150–510 base pairs. Samples with size calibration below 90 % were manually adjusted. An automatic panel editor was generated for each selective primer combination (Curtin et al. 2007) and then manually modified. For analysis, the matrices generated for each primer combination were combined into one matrix (Wooten and Tolley-Jordan 2009). Ten percent of the total individuals were replicated, the error rate being calculated as the ratio of number of fragment differences/total number of comparisons (Bonin et al. 2004).

Nine nuclear microsatellites markers were selected and isolated from *D*. *confertifolia* (Takayama et al. 2011) based on repeatability and scoring suitability. A total of 281 individuals of *D. confertifolia*, 13 of *D. andina,* 101 *of D. winteri* var*. chilensis*, and 22 of *D. winteri* var*. winteri* was analyzed. For fluorescent labeling of PCR amplified fragments, we used the 5′-tailed primer method (Boutin-Ganache et al. 2001) following Takayama et al. (2011). Different dyes (6-FAM, NED, PET, and VIC) for four combinations of multiplex PCR amplification were used, following a modified protocol of the Qiagen Multiplex PCR Kit (Qiagen, Hilden, Germany). Four multiplex PCR reactions were done as follows: AWU5A, AOH4B with 6-FAM, A3340, ATEAE with VIC, AWL0 W, A9194, AX48Q with NED, BDYVU, AOES3 with PET. Each reaction was performed in a final volume of 3 µL containing 0.2 μM of each reverse primer, 0.04 μM of each forward primer, and 0.6 μM of fluorescent dye-labeled primer. Touchdown thermal cycling programs were used as follows: initial denaturation at 95 °C for 5 min, followed by 20 cycles of denaturation at 95 °C for 30 s, annealing at 63 °C for 90 s (decreased 0.5 °C per cycle), and extension at 72 °C for 60 s, plus 25 cycles of denaturation at 95 °C for 30 s, annealing at 55 °C for 90 s, and extension at 72 °C for 60 s; a final extension step was performed at 60 °C for 30 min. The amplified fragments were run with a size standard (GeneScan 600, Applied Biosystems, Foster City, CA, USA) on an automated sequencer (ABI 3130xl). GeneMarker ver. 1.85 (SoftGenetics LLC) was used for scoring.

### Data analysis

For AFLPs the program ARLEQUIN 3.5.1.2 (Excoffier et al. 2005) was used for calculating the total number of different phenotypes in each population and the average gene diversity over loci (AGDOL; the probability that two homologous band sites, randomly chosen, are different). The estimations of other genetic diversity parameters by populations, i.e., percentage of polymorphic bands (PPB), total number of AFLP bands (TNB), and Shannon Diversity Index (SDI) (*H*_Sh_ = −Σ [*p*_*i*_ ln(*p*_*i*_)] where *p*_*i*_ is the frequency of the *i*th band in the respective population based on all AFLP bands recorded) were performed using FAMD ver. 1.25 (Schlüter and Harris 2006).

With respect to genetic divergence parameters and population structure, the number of private bands (NPB) was calculated with FAMD ver. 1.25 (Schlüter and Harris 2006), and the Rarity Index (RI) (Schönswetter and Tribsch 2005) was estimated with R-script AFLPdat (Ehrich 2006). Pairwise *F*_ST_ were calculated according to Weir and Cockerham (1984), and the probabilities of random departure from zero for obtaining *F*_ST_ were calculated using the exact test with 10,000 permutations in ARLEQUIN 3.5.1.2. (Excoffier et al. 2005). A NeighborNet algorithm (Bryant and Moulton 2004), implemented by the software SplitsTree4 ver. 4.10 (Huson and Bryant 2006), was executed using a Nei-Li distance matrix calculated from the original AFLP matrix. An analysis of molecular variance (AMOVA) with ARLEQUIN 3.5.1.2 (Excoffier et al. 2005) was implemented for estimating genetic differentiation among and within populations (hierarchical structuring). For calculation of probabilities, 1,023 permutations were used, which reveals significance of the variance components. For appraisement of population structure (with a Bayesian clustering method), the program STRUCTURE 2.3.3 (Falush et al. 2007; Hubisz et al. 2009; Pritchard et al. 2000) was employed. To assign individuals into *K* clusters, an admixture model with correlated allele frequencies (Falush et al. 2003) was used. The number of steps was 100,000, with 50,000 iterations, and 10 replicate runs in each *K* from 1 to 10. The highest level of structure was deduced from a posterior probability of the data for a given *K* and ∆*K* value (Evanno et al. 2005). A Pearson correlation was used to test correlation between values of genetic diversity and genetic divergence, and a Mann–Whitney *U* test was performed to compare means of independent samples for the previous parameters. In both cases the program SPSS ver. 15.0 (©SPSS Inc.) was used.

For microsatellites the program GENEPOP 4.0 (Raymond and Rousset 1995) was used to test linkage disequilibrium (LD) and significant deviation from Hardy–Weinberg equilibrium (HWE) between loci in each population (Markov chain method 10,000 dememorisation steps, 1,000 batches, 500 iterations per batch). The frequency of null allele in each marker was calculated following Brookfield (1996) using Micro-Checker 2.2.3 (van Oosterhout et al. 2004). The genetic diversity parameters for each species and population, allelic richness (*A*_R_), expected proportion of heterozygotes (*H*_E_), number of alleles per locus (*N*_A_), and inbreeding coefficient (*F*_IS_), were calculated using FSTAT 2.9.3.2 (Goudet 1995). GENALEX 6 (Peakall and Smouse 2006) was used to estimate the genetic divergence parameters, number of private alleles (*N*_PA_), and number of locally common alleles (*N*_LCA_). Allelic richness was calculated for populations that contained seven or more individuals using the rarefaction method (Hurlbert 1971). The genetic relationships among populations were evaluated by constructing a neighbor-joining tree based on *D*_A_ genetic distance (Nei et al. 1983) using the program Populations 1.2.30 (Langella 1999). Pairwise *F*_ST_, AMOVA, STRUCTURE, and Pearson correlation were estimated in the same way as with AFLP.

The direction of migration between island populations of *D. confertifolia* was inferred by using microsatellite data based on the log Bayes factor (*LBF*) as per the model ranking method (Beerli and Palczewski 2010) using the coalescent based MCMC method implemented in Migrate-3 (Beerli and Felsenstein 1999, 2001). The *LBF*s based on the Bézier approximation score and harmonic mean were estimated using microsatellite data. We compared the null model of no migration (*M*_0_) to the models of unidirectional migration from Alejandro Selkirk to Robinson Crusoe Island (*M*_1_) and vice versa (*M*_2_). In order to standardize population sizes, the 16 populations of *D. confertifolia* in Robinson Crusoe Island and 15 populations in Alejandro Selkirk Island were pooled each and randomly sampled for each replicate (50 individuals from each region). Runs were carried out under a Brownian model multiple times with varying parameter settings to achieve convergence, and for the final MCMC parameters were one long chain with 200,000 recorded steps and 100-step increment with a burn-in of 10,000. Uniform priors (minimum, maximum, and delta) were placed for both theta (0, 20, and 2) and *M* (0, 20, and 2). Ten replicates of single long Markov chains were implemented using different random number seeds. The MIGRATE analysis was performed at the University of Oslo Bioportal (https://www.bioportal.uio.no/).

## Results

### AFLPs

Among the species of *Drimys*, we found a total of 583 fragments, of which 574 are polymorphic (98.5 %, Table 1). In *D*. *confertifolia* the total number of fragments is 576 (100 % polymorphic bands), in *D*. *andina* 383 fragments (373 bands polymorphic, 97.4 %), and *D*. *winteri* 485 fragments (438 polymorphic, 90.3 %, Table 1).

With each pair of primers, the numbers of fragments in each species (*D. confertifolia/D. andina/D. winteri*) are: 153/81/126/for primers *Mse*I–CTG/*Eco*RI–ACA (FAM); 120/76/102 for *Mse*I–CAC/*Eco*RI–ATG (VIC); 112/72/80 for *Mse*I–CTC/*Eco*RI–ACA (FAM); 104/58/76 for *Mse*I–CAG/*Eco*RI–AAG (VIC); and 77/42/54 for *Mse*I–CTC/*Eco*RI–AGC (NED). All individuals had unique AFLP phenotypes. The reproducibility of the bands was 95 %.

### Microsatellites

All microsatellite markers, except for one marker AOES3, were successfully genotyped in 281 individuals of *D. confertifolia*, 123 of *D. winteri*, and 13 of *D. andina.* The marker AOES3 resulted in no amplification in two populations of *D. confertifolia*, and several populations of *D. winteri* and of *D. andina*. Hence, we used the remaining eight markers for further population analyses. An exact test for HWE across populations and loci showed 14 of 248 in *D. confertifolia*, six of 80 in *D. winteri*, and one of eight in *D. andina* deviating from HWE with a positive *F*_IS_, (*P* < 0.05) after Bonferroni correction. The frequency of null alleles across populations and loci was estimated using Micro-Checker, resulting in the highest frequency of 0.188 (AWL0W), with an average frequency of 0.080 in all of the eight markers. Significant LD was not found between any pairwise combination of loci in all populations within each species (*P* < 0.05) after Bonferroni correction.

### Genetic diversity

The AFLP measures of genetic diversity in populations of *Drimys* analyzed are shown in Table 1. The average estimates of genetic diversity in *D. confertifolia*, from Robinson Crusoe/Alejandro Selkirk Islands, are for percentage of polymorphic bands (PPB) 66.8/58.8; for total number of bands (TNB) 433.5/414.7; for Shannon Diversity Index (SDI) 103.98/90.53; and for average genetic diversity over loci (AGDOL) 0.24/0.22. All estimates reveal a similar pattern and are highly correlated with *r* ranging from 0.897 to 0.956 (*n* = 22, *P* < 0.001). This pattern holds when those correlations on the single islands were tested. Comparisons of genetic diversity between populations on the northern side of Robinson Crusoe Island (1–7) with populations on the southern side (9–16) show significant differences in the parameters TNB, SDI, and AGDOL. In Alejandro Selkirk Island, the northern populations (17, 18, 23) are not significantly different in all genetic diversity parameters from the southern populations (28–31). Overall the genetic diversity within populations is higher on Robinson Crusoe Island (average SDI 103.98 ± 6.88) than on Alejandro Selkirk Island (average SDI 90.53 ± 10.99).

For each of the species, the total genetic variation (AGDOL) was 0.276 in *D. confertifolia*, 0.203 in *D. andina*, and 0.208 in *Drimys winteri*. In *D. confertifolia* a higher value was found on Robinson Crusoe Island (0.259) compared to Alejandro Selkirk Island (0.234) (Table 2).

**Table 2:** Genetic diversity with AFLP and microsatellites for *Drimys confertifolia*, *D. winteri*, and *D. andina*

Species	AFLP	Microsatellites
	AGDOL	*H*_E_
*D. confertifolia* Robinson Crusoe Island	0.259	0.685
*D. confertifolia* Alejandro Selkirk Island	0.234	0.509
*D. confertifolia* (combined)	0.276	0.675
*D. winteri*	0.208	0.733
*D. andina*	0.203	0.501

*AGDOL* average gene diversity over loci, *H*_*E*_ expected proportion of heterozygotes

In microsatellites, the values of genetic diversity parameters for all species of *Drimys* are shown in Table 1. The average values in *D*. *confertifolia* from Robinson Crusoe/Alejandro Selkirk Islands for number of alleles per locus (*N*_A_) are 4.89/3.73; for allelic richness (*A*_R_) are 4.24/3.30; and the expected proportion of heterozygotes (*H*_E_) are 0.62/0.47. All estimates are correlated; *H*_E_ was highly correlated with *N*_A_ (*r* = 0.868) [*n* = 22, *P* = 0.000], and *A*_R_ (*r* = 0.634) [*n* = 22, *P* = 0.002]. When analyzing this parameter by island, only the pair *N*_A_ and *H*_E_ is correlated (*r* = 0.810) [*n* = 15, *P* = 0.000] in Robinson Crusoe Island; no correlations were found between these three parameters in Alejandro Selkirk island. Positive *F*_IS_ values (*P* < 0.05) after Bonferroni correction were significant in 11 populations of *D. confertifolia* and in four populations of *D. winteri*.

When dividing the populations of Robinson Crusoe Island into northern (1–7) and southern (9–16) sections, they show no significant difference. The same analysis holds for Alejandro Selkirk Island, revealing no significant differences between the parameters *N*_A_, *A*_R_, and *H*_E_, among northern (17, 18, 23) and southern populations (28–31).

The genetic diversity (*H*_E_) within species was 0.675 in *D*. *confertifolia*, 0.733 in *D*. *winteri*, and 0.501 in *D*. *andina* (Table 2). Calculating this value for *D. confertifolia* on each of the two islands revealed 0.685 on Robinson Crusoe Island and 0.509 on Alejandro Selkirk Island.

### Genetic divergence

For AFLPs, the values of genetic divergence are shown in Table 1. In *Drimys confertifolia* the number of private bands (NPB) and rarity index (RI) in Robinson Crusoe/Alejandro Selkirk Islands are 0.40/0.14 and 1.50/1.76 respectively. These values are not correlated, (*r* = 0.065) [*n* = 22, *P* = 0.775]. The same lack of correlation occurs when analyzing Robinson Crusoe and Alejandro Selkirk Islands separately. Comparison between northern populations with southern populations on Robinson Crusoe Island reveals significant differences only for the Rarity Index. The same analysis in Alejandro Selkirk Island reveals no significant differences in both measures of divergence.

Pairwise *F*_ST_ values between *D. confertifolia* on the two islands and continental *D. andina* and *D. winteri* reveal the highest differentiation (*F*_ST_ = 0.357) between *D. winteri* and *D. confertifolia* of Alejandro Selkirk island, and the lowest between *D. confertifolia* from Robinson Crusoe and Alejandro Selkirk (*F*_ST_ = 0.186) (Table 3). The mean *F*_ST_ value among populations within *D. confertifolia* was 0.141 and 0.212 in *D. winteri*. A high proportion of pairwise *F*_ST_ values were significant in *D*. c*onfertifolia*, and all values were significant in *D*. *winteri* (Table S1, S2).

**Table 3:** *F*_ST_ values among *Drimys confertifolia* in Robinson Crusoe and Alejandro Selkirk Island, *D. andina*, and *D. winteri*

	*D. confertifolia* (RC)	*D. confertifolia* (AS)	*D. andina*	*D. winteri*
*D. confertifolia* (RC)		0.186	0.204	0.266
*D. confertifolia* (AS)	0.150		0.263	0.357
*D. andina*	0.245	0.283		0.203
*D. winteri*	0.060	0.101	0.179	

Above diagonal are estimates from AFLP data, below diagonal from microsatellites

*RC* Robinson Crusoe Island, *AS* Alejandro Selkirk Island

For microsatellites, the parameters for estimating genetic divergence within *Drimys* species are shown in Table 1. In *D. confertifolia* the number of private alleles (*N*_PA_) and number of locally common alleles (*N*_LCA_) in Robinson Crusoe/Alejandro Selkirk Islands are 0.04/0.02 and 0.75/0.50, respectively. The differences between the values of this parameter are not correlated (*r* = 0.361) [*n* = 22, *P* = 0.099]. The same tendency is seen when we analyzed separately each of the islands. We also compare the *N*_PA_ between the two island populations of *D. confertifolia*. The *N*_PA_ of Robinson Crusoe populations is 3.125 (±0.875), and that of Alejandro Selkirk is 0.75 (±0.366). Geographical division between northern (17, 18, 23) and southern populations (28–31) in Alejandro Selkirk Island showed no significant differences. The same lack of divergence between northern and southern populations also occurs on Robinson Crusoe Island.

The highest values of *F*_ST_ between *D. confertifolia* in Robinson Crusoe and Alejandro Selkirk Island and the other species of *Drimys* correspond to the pair *D. confertifolia* (Alejandro Selkirk island)–*D. andina* (*F*_ST_ = 0.283), and the lowest to *D. confertifolia* (Robinson Crusoe)–*D. winteri* (*F*_ST_ = 0.060) (Table 3). In *D*. *confertifolia* the mean *F*_ST_ value among populations was 0.109, and in *D*. *winteri* 0.139. The pairwise genetic differentiation values were significant in all species under study (Table S1, S2).

### Genetic structure

Results of the analysis of molecular variance (AMOVA) at different hierarchical levels in *D*. *confertifolia* are shown in Table 4. A high percentage of variation was found within populations, with values of 76 % for AFLPs and 50 % for microsatellites. Among populations within the islands, the values for AFLPs and microsatellites were low and 6 and 5 %, respectively, whereas a high percentage of variation between Robinson Crusoe and Alejandro Selkirk Islands was found to be 18 and 15 %, respectively.

**Table 4:** Summary of analyses of molecular variance (AMOVA) for AFLPs and microsatellites in *Drimys confertifolia*

Source of variation	AFLPs				Microsatellites			
	*df*	SS	Variance components	Total variance (%)	*df*	SS	Variance components	Total variance (%)
Between islands	1	1925.61	16.19	17.97	1	102.7	0.4	14.61
Among populations within island	20	2,592.67	5.22	5.79	20	119.2	0.2	5.09
Within populations	237	16,278.05	68.68	76.24	500	1,194.8	2.4	50.30

The total variance contributed by each component (%), and its associated significance (*n* = 1,023 permutations) are shown

*df* degrees of freedom, *SS* sum of squares

**Fig. 3:**
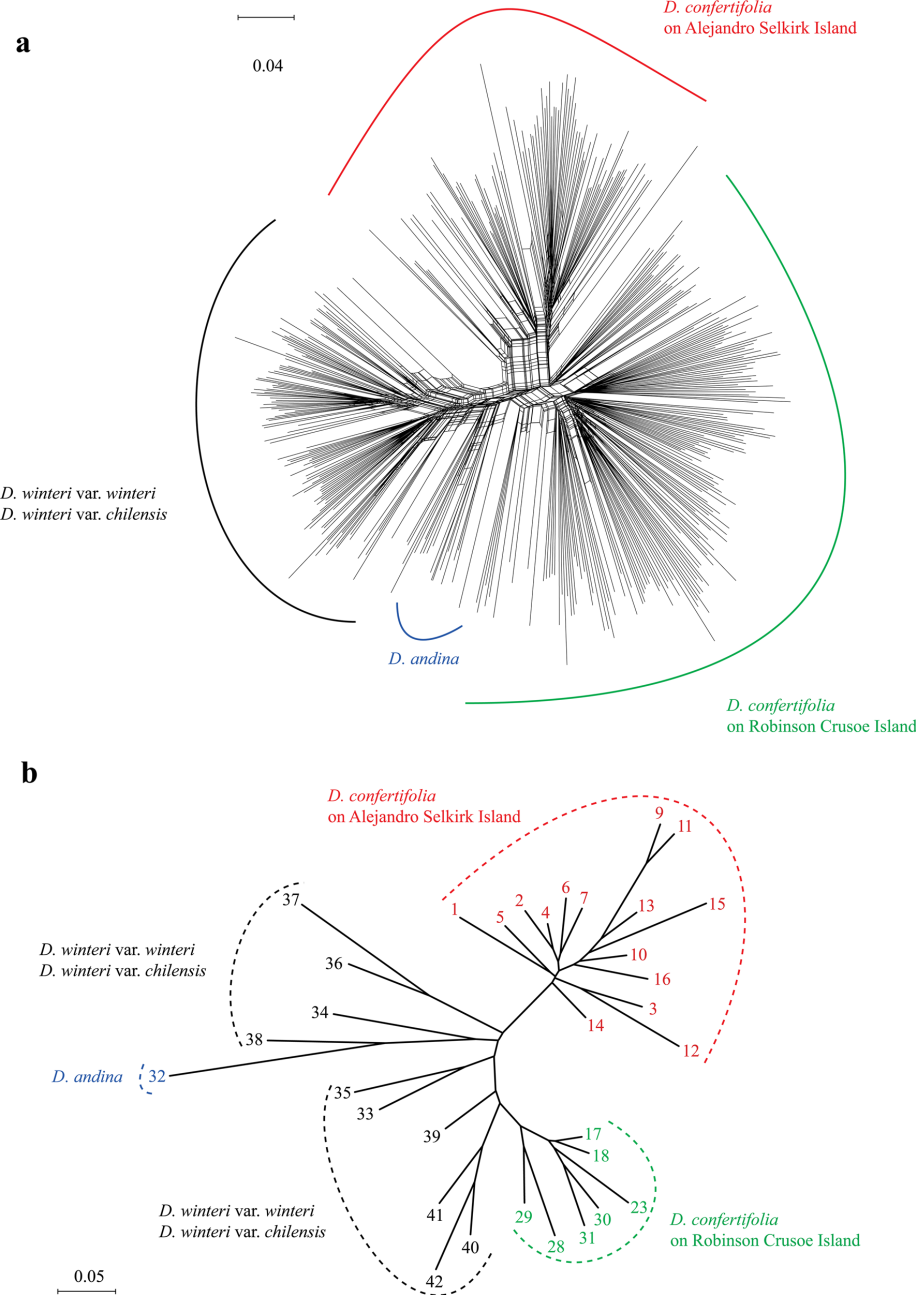
Genetic relationships among populations of *Drimys*. SplitsTree NeighbourNet (phylogenetic network) of AFLP data showing relationships among individuals of *D. andina*, *D. confertifolia*, *D. winteri* var. *chilensis* and *D. winteri* var. *andina* (**a**), and Neighbour-Joining tree based on microsatellites showing relationships among populations in the same species of *Drimys* (**b**)

The NeighbourNet tree based on AFLP data using all individuals of *D*. *confertifolia*, *D*. *andina*, and *D*. *winteri* is shown in Fig. 3a. A separation between species is observed with *D. confertifolia* forming two groups, the first clearly differentiated and corresponding to Alejandro Selkirk Island, and the second to the populations of Robinson Crusoe Island. No clear separation between populations is visible, with only a weak divergence of populations 23, 30, 31 on Alejandro Selkirk Island and population 13 on Robinson Crusoe Island. The cluster of *D*. *winteri* does not show a separation between variety *winteri* and var. *chilensis*. No geographical partitioning is observed in all species.

The Neighbour-Joining tree using microsatellite data at the population level is shown in Fig. 3b. *Drimys* *confertifolia* forms two groups, each restricted to a different island. The populations from Alejandro Selkirk Island are linked more closely to *D.* *winteri* than to the other populations on Robinson Crusoe Island. No geographical division is found in *D*. *winteri*, and no division between *D*. *winteri* var. *winteri* and *D*. *winteri* var. *chilensis* is seen. *Drimys* *andina* is connected to *D*. *winteri*.

**Fig. 4:**
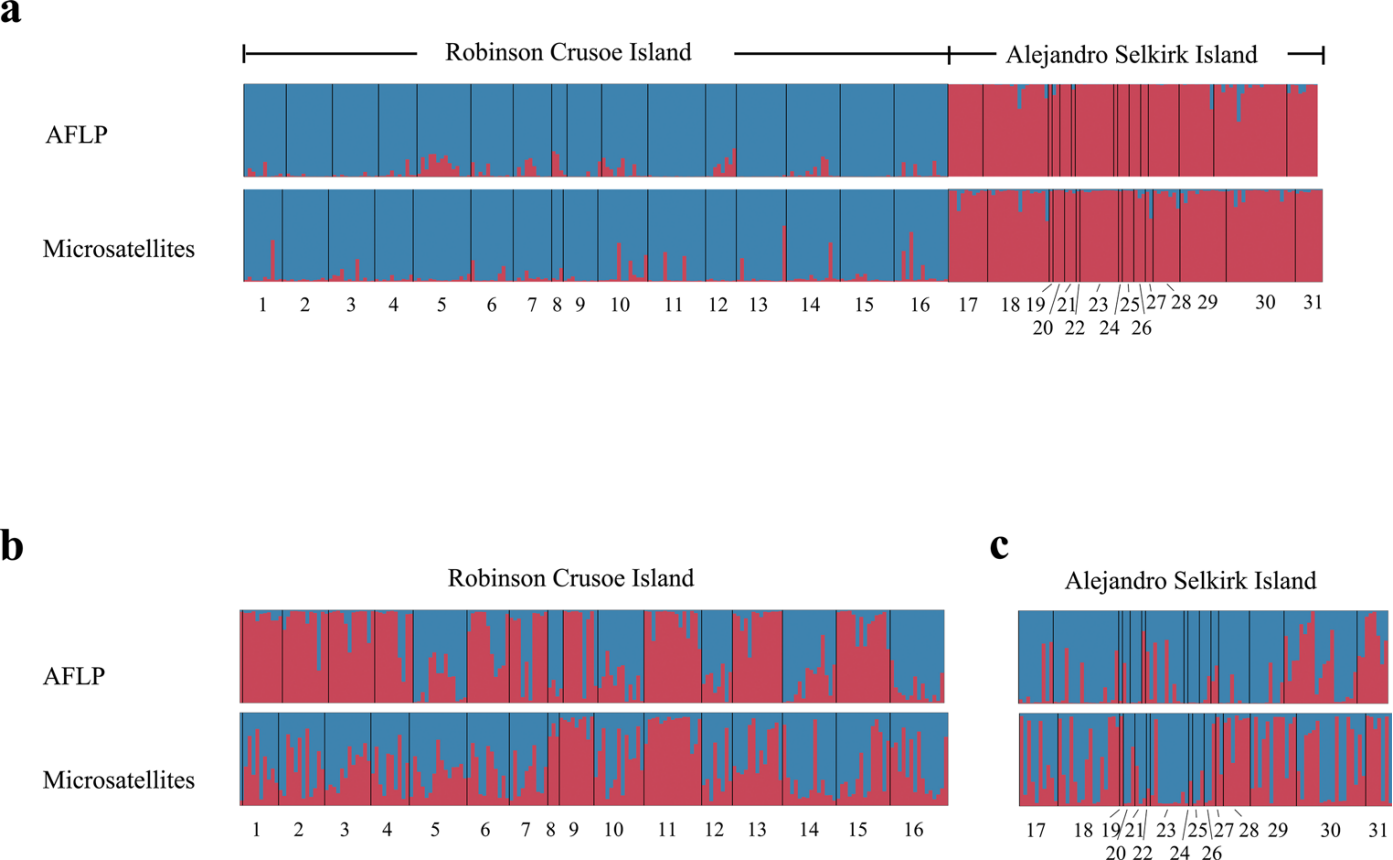
Genetic affinities from AFLP and microsatellite data among populations of *Drimys confertifolia* on both islands (**a**), on Robinson Crusoe Island (**b**), and Alejandro Selkirk Island (**c**) by means of STRUCTURE (all *K* = 2)

Testing for genetically coherent groups in *D. confertifolia* using the Bayesian approach STRUCTURE 2.3.3 (Fig. 4), the group number *K* = 2 explained best the groupings found by both AFLPs and microsatellites and revealed a very low degree of admixture among the islands. Analyzing each of the islands separately, *K* = 2 again best explains the grouping for both molecular markers, however, with a high proportion of individuals showing strong admixture.

Mantel tests between genetic differentiation (*F*_ST_/(1 − *F*_ST_)) (Rousset 1997) and geographical distance did not reveal any significant correlation for the *D*. *confertifolia* populations. The tests for Robinson Crusoe Island provided *r* ^2^ = 0.0344 and *r* ^2^ = 0.0036 for AFLP and microsatellites, respectively. For Alejandro Selkirk the values were *r* ^2^ = 0.0333 for AFLP and *r* ^2^ = 0.0080 for microsatellites.

Results of the MIGRATE analyses (Table 5) showed consistent results with smooth histograms of theta and *M*, suggesting that the Markov chain had converged on the stationary distribution. The both LBFs based on the Bézier approximation score and the harmonic mean constantly indicate that two models of migration (*M*_1_ and *M*_2_) are more likely than the null hypothesis of no gene flow (*M*_0_) between the two islands (Table 5). The *LBF*s are higher in a unidirectional migration model from Robinson Crusoe to Alejandro Selkirk Island (*M*_2_) than the other model involving arrival first to Alejandro Selkirk and then migration to Robinson Crusoe Island (*M*_1_).

**Table 5:** Results of comparison of different migration models between the two islands for *Drimys confertifolia*

Model	Bézier approximation score			Harmonic mean		Model rank
	ln (mL)	LBF	Model rank	ln (mL)	LBF	
M0: no migration	−313,478	284,888	3	−24,451	21,324	3
M1: migration from Alejandro Selkirk to Robinson Crusoe	−29,489	899	2	−5,894	2,767	2
M2: migration from Robinson Crusoe to Alejandro Selkirk	−28,590	0	1	−3,127	0	1

The model rank with 1 is the preferred model

*ln (mL)* log marginal likelihoods, LBF log Bayes factor [=ln (marginal likelihood of M0) − ln (marginal likelihood of Mn)]

## Discussion

### Evolutionary and taxonomic relationships

Within the complex of Chilean species of *Drimys*, some taxonomic changes have been suggested during past decades. *Drimys confertifolia* was early regarded as a variety of *D.* *winteri* (Johow 1896; Reiche 1895; Skottsberg 1921), but Smith (1943) later raised it to the rank of species. *Drimys andina* was also earlier considered a variety of *D. winteri* (Marticorena and Quezada 1985; Muñoz 1980; Smith 1943), but it, too, has been elevated to specific level (Rodríguez and Quezada 1991). In the most recent revision of the genus in Chile (Rodríguez and Quezada 2001), two varieties of *D. winteri* have been recognized, var. *winteri* and var. *chilensis*, based on morphological, geographical and ecological characteristics.

Our results from network and neighbor-joining tree analyses using AFLPs and microsatellites (Fig. 3) incorporate new information about Chilean species of *Drimys*. The populations of *D*. *confertifolia* on Alejandro Selkirk Island form a clade with those of *D*. *winteri*, and although our data for *D*. *andina* are minimal (one population), all individuals of this species formed a single group, positioned within the complex of *D*. *confertifolia*. The same trend was shown by Jara-Arancio et al. (2012) in studies with seven leaf characters, where *D*. *andina* and *D*. *confertifolia* are not clearly separated from *D*. *winteri*. Phytochemical characters, however, reveal the presence of essential oils and flavonoids in *D*. *andina* that differ from those found in *D*. *winteri* (Muñoz-Concha et al. 2004; Ruiz et al. 2002). Furthermore, the habit of *D*. *andina* is a compact shrub, rather than a large tree, and the arrangement, shape, and margin of leaves also differ (Rodríguez and Quezada 1991). Ruiz et al. (2008), using ITS markers, showed *D*. *andina* to be the most divergent species, similar to the molecular results of Jara-Arancio et al. (2012).

At the variety level, Smith (1943) and Rodríguez and Quezada (2001) used numbers of flowers, petals, and ovules, together with geographical distribution, to distinguish the varieties of *Drimys*. *Drimys winteri* var. *winteri* is restricted to the southern tip of Chile (Prov. Aysén until Prov. Antárctica Chilena), whereas *D. winteri* var. *chilensis* occurs through most of the country (North to South, i.e., Prov. Limari to Prov. Aysén). Our molecular results, however, do not support these varietal concepts within *D*. *winteri* (var. *winteri* and var. *chilensis*); the varieties are neither clearly differentiated nor geographically separated (Fig. 3). Ruiz et al. (2008) did not resolve relationships between these two varieties. Jara-Arancio et al. (2012), using RAPD data, showed three groups in *D. winteri*, but these do not correlate with the varietal distinctions, and the highest divergence (morphological and genetic) occurs from the populations of the coastal refugia of Fray Jorge and Talinay (Coquimbo region, Chile).

Regarding evolutionary relationships among the taxa of *Drimys* of Chile, *D. confertifolia* has been shown, based on cp DNA and ITS sequences (Marquínez et al. 2009), to be more distinct from *D*. *winteri* and *D*. *andina* than these two are from each other. With ITS and RAPDs each separately, however, *D. confertifolia* appears to link more closely with *D.* *andina* and not *D. winteri* (Jara-Arancio et al. 2012; Ruiz et al. 2008). Earlier studies based on morphology have uniformly concluded that the island species derived from, and hence would be closest to *D. winteri* (Bernardello et al. 2006; Rodríguez and Quezada 2001; Ruiz et al. 2002; Smith 1943). In part this view was supported because at that time most workers were treating *D. andina* as simply a variety of *D. winteri* (Muñoz 1980; Marticorena and Quezada 1985; Rodríguez et al. 1983; Smith 1943).

Within *D. confertifolia*, our molecular analyses reflect a clear separation between the populations from Robinson Crusoe and Alejandro Selkirk Islands by both markers (Fig. 3a, b). From a taxonomic point of view, however, no morphological differences have been detected between populations on the two islands (Smith 1943). It appears that after isolation on Alejandro Selkirk Island, these populations have began to diverge genetically, but differences in morphology are not yet observable–perhaps because the environment is not sufficiently different for directional selection to be imposed on the phenotype in this species.

### Migration routes to and within the Archipelago

In the case of *Drimys* of the Juan Fernández Archipelago it is assumed that the island populations have been derived from continental South America (Chile), rather than the reverse, for two reasons. First, the islands are geologically much younger (1–4 myr) than the continent (Stuessy et al. 1984), which suggests a stronger likelihood of the species originating on the continent, and dispersing to the islands. Second, South America contains seven species of *Drimys* (Smith 1943), suggesting a center of diversity of related species within the genus, and follows the standard idea that the greatest diversity is also frequently the center of origin (Barthlott et al. 2005). While it is not impossible for the reverse to be true (the islands as the ‘source’), it is less likely, and thus, it is reasonable as a null hypothesis to assume that the island species originated from out of the continental complex.

Numerous models have been proposed for colonization of oceanic islands. An intuitively obvious one is the “progression rule” (Funk and Wagner 1995), whereby the ancestral species is usually regarded as having arrived first on the oldest island, followed by colonization of new emerging islands as they become available. The ‘progression rule’, which was described in the introduction is certainly a reasonable biogeographic perspective, and many studies have, in fact, supported this hypothesis (Funk and Wagner 1995). Given the geology history of the Juan Fernández Archipelago, the progression rule would suggest that the original colonizing population(s) of *Drimys* were first established on Robinson Crusoe Island and later on Alejandro Selkirk Island. However, given the significant genetic differentiation between *Drimys* on the two islands, one might infer a long time since colonization, perhaps even to 1 million years, i.e., soon after the younger island was formed.

This inference of mode of migration can be tested with microsatellite data. Of the two possible migration models, i.e., model 2, migration from Robinson Crusoe to Alejandro Selkirk, and model 1, migration from Alejandro Selkirk to Robinson Crusoe Island (Table 5), the MIGRATE analysis using microsatellite data support propagule arrival on the older island (RC) and transfer from there to the younger island (AS). The lower values of genetic diversity (within populations and the island as a whole) in both AFLP and microsatellite data from Alejandro Selkirk Island in comparison to those from Robinson Crusoe Island are in accordance with this model 2 (Tables 1, 2). In addition, populations of Alejandro Selkirk Island contain a very low number of private alleles in contrast to Robinson Crusoe Island, suggesting recent immigration of Alejandro Selkirk populations. This pattern in the Juan Fernández Archipelago, i.e., migration from older to younger islands, is similar to that found in other taxa on Pacific islands, e.g., in *Plantago* (Plantaginaceae; Dunbar-Co et al. 2008), *Metrosideros* (Myrtaceae; Percy et al. 2008), or *Schiedea globosa* (Caryophyllaceae; Wallace et al. 2009) in the Hawaiian Islands.

As for mode of dispersal, the fruits of *Drimys* are fleshy berries, light violet to black at maturity, which might be attractive to birds. The presence of *Drimys* in the Juan Fernández Archipelago, therefore, might have resulted by dispersal from the continent by birds (zoochory), perhaps by transporting fruits internally (Bernardello et al. 2006). Annual production of seeds in *D. winteri* in the continent in a forest of the Coastal Cordillera in Valdivia (Chile) has been estimated by Donoso (1993) to range between 400,000–3,000,000 seeds per hectare, suggesting that there would have been no scarcity of seeds in continental progenitor populations. Although no specific bird migration routes are known between southern South America and the Juan Fernández Archipelago (Dorst 1962), some waterfowl from continental Chile have been recorded sporadically in these islands (Weller 1980). Once in the archipelago, presumably first on Robinson Crusoe Island, bird dispersal would again be implicated as a means of arriving on Alejandro Selkirk Island. Long distance dispersal for *D. confertifolia* is probably not a limitation, therefore, but establishment seems to be a challenge. Studies of *D. confertifolia* by Cuevas and Figueroa (2007) reveal seed banks on Robinson Crusoe Island. However, they did not observe any germination of seeds under greenhouse conditions even after 9 months when soil from the *Drimys* forest was used (Cuevas and Figueroa 2007). These authors suggested the possibility of breaking seed dormancy by passage through the digestive tract of the birds, but no evidence exists to support this contention.

### Genetic consequences of anagenetic speciation

Numerous factors influence levels of genetic variation within and among populations on oceanic islands (Stuessy et al. 2013). Here we consider three that in our judgment best explain patterns of genetic variation within *D*. *confertifolia*. The first factor is the geography and age of the island. Stuessy et al. (1998) have estimated a loss of surface of approximately 95 % for Robinson Crusoe Island in the last four million years, with only 28 % reduction for Alejandro Selkirk during the past 1–2 million years. Oceanic islands are composed mainly of volcanic ash and lava, which erode rapidly due to wind and water, especially wave action. Islands are also continually subsiding on the plates from which they originated. Over millions of years, these factors combine to reduce the size of oceanic islands. Loss of surface area would be accompanied by diminution of types of habitats, and a compaction of the flora, resulting in the potential for gene flow among populations. The absence of geographical structure in genetic variation in *D. confertifolia* in Robinson Crusoe Island (Fig. 3), therefore, may reflect loss of habitats. Another possibility, however, is that there never was reduction in gene flow, given that the species is likely wind pollinated (Bernardello et al. 2001), with possibly continuous gene flow during the history of the island. In the younger Alejandro Selkirk Island, geographical partitioning of genetic diversity is also not seen, but in this case it may be due to more recent arrival of *Drimys* to the island, and hence, insufficient time for genetic differentiation to have developed among populations. Multiple origins cannot be rejected based on our molecular data, but the morphological unity within the islands and the consistent differences in contrast with continental species supports the concept that island populations developed from only a single introduction.

Biological characteristics can also influence levels of genetic variation within and among populations of oceanic islands. *Drimys confertifolia* has white-yellow, hermaphroditic flowers, and is protogynous, all of which suggest insect pollination. The flowers are without nectar, however, and no floral visitors have been detected (Anderson et al. 2001; Bernardello et al. 2001). There are, in fact, no known insect pollinators documented for the Juan Fernandez Islands (Anderson et al. 2001; Bernardello et al. 2001). Consequently, these same authors have inferred a pollination system by wind. Investigations on *D. granadensis* from the continent have shown it to have a generalist insect pollination spectrum (Marquínez et al. 2009). If wind pollination for *Drimys* in the islands is, in fact, correct, then this characteristic would facilitate gene exchange among individuals and populations. Another biological feature to be considered is population size. For some reason, *D. confertifolia* has larger populations and more individuals on Robinson Crusoe Island than on Alejandro Selkirk Island. Pollen profiles covering the Holocene on Alejandro Selkirk Island (Haberle 2003) show little variation in the abundance of *D. confertifolia* pollen indicating consistently small populations in contrast to e.g., *Coprosma* and *Pernettya*. Pollen abundance of the latter two genera has obviously reacted significantly to oscillations between dry and wet climatic conditions (Haberle 2003). In total, Ricci (1992) estimated approximately 1000 existing individuals of *D. confertifolia* on Alejandro Selkirk, 20 % of the level she estimated for Robinson Crusoe Island. The largest population on Alejandro Selkirk Island from our observations and collections in 2011 was 19 individuals, compared with Robinson Crusoe Island where we observed up to 200 plants per population. Vargas et al. (2010) estimated a density of up to 60 trees/ha of *D. confertifolia* in the Robinson Crusoe Island. If effective population size is small, then genetic drift is obviously a factor. Also, long generation times combined with small effective population sizes would promote drift and prevent rapid build-up of diversity.

A third factor, and one that we emphasize in this paper, is mode of speciation. Arrival and establishment of individuals on an oceanic island will obviously occur with only a few founding individuals (Nei et al. 1975), resulting in a reduction of genetic diversity in the founding population, i.e., a founder effect (Austerlitz et al. 2000; Caetano et al. 2012). After establishment, the population slowly increases in size and also presumably in genetic variation as a result of mutation and recombination. Dispersal of propagules to distinct habitats starts the process of splitting, or cladogenesis, whereas dispersal to similar habitats does not impose such stringent selection. The end result after millions of years is speciation either by cladogenesis or anagenesis (or, of course, also extinction). In cladogenesis, the number of endemic species will increase, but genetic variation within each will remain low. In anagenetic speciation, however, an ancestral lineage is transformed into a single new species, and it will have accumulated levels of genetic variation over generations that are similar to or even exceed those of the progenitor species (Stuessy et al. 2006; Stuessy 2007).

As *Drimys confertifolia* is the only endemic species of its genus in the Juan Fernández Archipelago, and excluding complex hypotheses involving extinction (Marquínez et al. 2009), the mode of speciation for this species must have been anagenetic. *D. confertifolia* exhibits a level of genetic variation similar to that in the parental species *D. winteri* from the continent (Table 2), being slightly lower or higher depending on the molecular marker used. The absence of geographical structure of genetic variation in both Robinson Crusoe and Alejandro Selkirk Islands supports these attributes for this type of speciation as hypothesized previously (Stuessy 2007). In the absence of adaptive radiation in diverse habitats, populations disperse over the ecologically uniform island and accumulate genetic variation via mutation and recombination. Over time, this leads to relatively high levels of genetic variation within anagenetically derived species.

Previous molecular studies in other endemic species that have originated by anagenesis in oceanic islands have revealed similar genetic patterns. Investigations by Pfosser et al. (2006) on *Dystaenia ibukiensis* (Apiaceae) from Japan, in comparison with the anagenetic derivative in Ullung Island, *D. takesimana*, showed a higher genetic diversity (AFLPs) in the endemic island species than in the one from Japan. Takayama et al. (2011) showed a slightly lower genetic diversity with nuclear microsatellites in the anagenetically derived *Acer okamotoanum* in Ullung Island in comparison with the progenitor *A. mono*. An AFLP study in this same pair of species by Pfosser et al. (2006), revealed a loss of alleles in *A.* *okamotoanum* in comparison with the parental species. Yamada and Maki (2012) showed in *Weigela coraeensis* var. *fragans* (Izu Island, Japan), in comparison with the progenitor *W. coraeensis* var. *coraeensis*, an incomplete anagenetic speciation process and a lower level of genetic diversity in the variety that occurs on the island. This is perhaps due to more recent immigration and less time available for accumulation of differentiating genetic variation. The other notable component of anagenetic speciation is that no geographical structure was observed in genetic variation among populations distributed over the island landscape. All indicators, the low proportion of variation among populations within the island (AMOVA: 5.79 %) (Table 4), the Mantel test (no correlation between genetic and geographical distance), and the patterns of the NeighbourNet are in line with the lack of geographical structure in *D. confertifolia* (Fig. 3). In addition, higher genetic variation within populations resembles the patterns seen in *Myrceugenia fernandeziana* and *M. schulzei* (López-Sepúlveda et al. 2013), both anagenetically derived species also endemic to the Juan Fernández Archipelago.

## Electronic supplementary material

Below is the link to the electronic supplementary material.

Supplementary material 1 (DOC 154 kb)

Supplementary material 2 (DOC 36 kb)

## References

[CR1] Anderson GJ, Bernardello G, Stuessy TF, Crawford DJ (2001). Breeding system and pollination of selected plants endemic to Juan Fernández Islands. Am J Bot.

[CR2] Austerlitz F, Mariette S, Machon N, Gouyon PH, Godelle B (2000). Effects of colonization processes on genetic diversity: differences between annual plants and tree species. Genetics.

[CR3] Baldwin BG, Crawford DJ, Francisco-Ortega J, Kim S-C, Sang T, Stuessy TF, Soltis DE, Soltis PS, Doyle JJ (1998). Molecular phylogenetic insights on the origin and evolution of oceanic island plants. Molecular systematics of plants II: DNA sequencing.

[CR4] Ballemain E, Ricklefs RE (2008). Are islands the end of the colonization road?. Trends Ecol Evol.

[CR5] Barthlott W, Mutke J, Rafiqpoor D, Kier G, Kreft H (2005). Global centers of vascular plant diversity. Nova Act Leopold.

[CR6] Beerli P, Felsenstein J (1999). Maximum-likelihood estimation of migration rates and effective population numbers in two populations using a coalescent approach. Genetics.

[CR7] Beerli P, Felsenstein J (2001). Maximum likelihood estimation of a migration matrix and effective population sizes in *n* subpopulations by using a coalescent approach. Proc Natl Acad Sci USA.

[CR8] Beerli P, Palczewski M (2010). Unified framework to evaluate panmixia and migration direction among multiple sampling locations. Genetics.

[CR9] Bernardello G, Anderson GJ, Stuessy TF, Crawford DJ (2001). A survey of floral traits, breeding systems, floral visitors, and pollination systems of the angiosperms of the Juan Fernández Islands (Chile). Bot Rev (London).

[CR10] Bernardello G, Anderson GJ, Stuessy TF, Crawford DJ (2006). The angiosperm flora of the Archipelago Juan Fernandez (Chile): origin and dispersal. Canad J Bot.

[CR11] Böhle UR, Hilger HH, Martin WF (1996). Island colonization and evolution of the insular woody habit in *Echium* L. (Boraginaceae). Proc Natl Acad Sci USA.

[CR12] Bonin A, Bellemain E, Eidesen PB, Pompanon F, Brochmann C, Taberlet P (2004). How to track and assess genotyping errors in population genetics studies. Mol Ecol.

[CR13] Boutin-Ganache I, Raposo M, Raymond M, Deschepper CF (2001). M13-tailed primers improve the readability and usability of microsatellite analyses performed with two different allele-sizing methods. Biotechniques.

[CR14] Brookfield JFY (1996). A simple new method for estimating null allele frequency from heterozygote deficiency. Mol Ecol.

[CR15] Bryant D, Moulton V (2004). Neighbor-Net: an agglomerative method for the construction of phylogenetic networks. Mol Biol Evol.

[CR16] Caetano S, Currat M, Pennington RT, Prado D, Excoffier L, Naciri Y (2012). Recent colonization of the Galapagos by the tree *Geoffroea spinosa* Jacq. (Leguminosae). Mol Ecol.

[CR17] Carine MA, Russell SJ, Santos-Guerra A, Francisco-Ortega J (2004). Relationships of the Macaronesian and Mediterranean floras: molecular evidence for multiple colonizations into Macaronesia and back-colonization of the continent in *Convolvulus* (Convolvulaceae). Am J Bot.

[CR18] Carlquist S (1974). Island biology.

[CR19] Cowie RH, Holland BS (2006). Dispersal is fundamental to biogeography and the evolution of biodiversity on oceanic islands. J Biogeogr.

[CR20] Crawford DJ, Stuessy TF, Iwatsuki K, Raven P (1997). Plant speciation on oceanic islands. Evolution and diversification of land plants.

[CR21] Crawford DJ, Sang T, Stuessy TF, Kim S-C, Silva M, Stuessy TF, Ono M (1998). *Dendroseris* (Asteraceae: Lactuceae) and *Robinsonia* (Asteraceae; Senecioneae) on the Juan Fernandez Islands: similarities and differences in biology and phylogeny. Evolution and speciation of island plants.

[CR22] Cuevas JG, Figueroa JA (2007). Seed germination of species of the Juan Fernández archipiélago under laboratory conditions. Gayana Bot.

[CR23] Curtin CD, Bellon JR, Henschke PA (2007). Genetic diversity of *Dekkera bruxellensis* yeast isolated from Australian wineries. FEMS Yeast Res.

[CR24] Donoso C (1993) Bosques templados de Chile y Argentina. Variación, estructura y dinámica. Editorial Universitaria, Santiago

[CR25] Dorst J (1962). The migration of birds.

[CR26] Drake DR, Mulder CP, Towns DR, Daugherty CH (2002). The biology of insularity: an introduction. J Biogeogr.

[CR27] Dunbar-Co S, Wieczorek AM, Morden CW (2008). Molecular phylogeny and adaptive radiation of the endemic Hawaiian *Plantago* species (Plantaginaceae). Am J Bot.

[CR28] Ehrendorfer F, Silberbauer-Gottsberger I, Gottsberger G (1979). Variation on the population, racial, and species level in the primitive relic angiosperm genus *Drimys* (Winteraceae) in South America. Plant Syst Evol.

[CR29] Ehrich D (2006). AFLPdat: a collection of R functions for convenient handling of AFLP data. Mol Ecol Notes.

[CR30] Eliasson U (1974) Studies in Galápagos plants. XIV. The genus *Scalesia* Arn. Opera Botanica 36:1–117

[CR31] Emerson BC (2002). Evolution on oceanic islands: molecular phylogenetic approaches to understanding pattern and process. Mol Ecol.

[CR32] Evanno G, Regnaut S, Goudet J (2005). Detecting the number of clusters of individuals using the software STRUCTURE: a simulation study. Mol Ecol.

[CR33] Excoffier L, Laval G, Schneider S (2005). Arlequin (version 3.0): an integrated software package for population genetics data analysis. Evol Bioinform Online.

[CR34] Falush D, Stephens M, Pritchard JK (2003). Inference of population structure using multilocus genotype data: linked loci and correlated allele frequencies. Genetics.

[CR35] Falush D, Stephens M, Pritchard JK (2007). Inference of population structure using multilocus genotype data: dominant markers and null alleles. Mol Ecol Notes.

[CR36] Funk VA, Wagner WL, Wagner WL, Funk VA (1995). Biogeographic patterns in the Hawaiian Islands. Hawaiian biogeography: Evolution on a hot spot archipelago.

[CR37] Gillespie RG, Roderick GK (2002). Arthropods on islands: colonization, speciation, and conservation. Ann Rev Entomol.

[CR38] Givnish TG, Millam KC, Mast AR, Paterson TB, Theim TJ, Hipp AL, Henss JM, Smith JF, Wood KR, Systma KJ (2009). Origin, adaptive radiation and diversification of the Hawaiian lobeliads (Asterales: Campanulaceae). Proc Roy Soc Biol Sci Ser B.

[CR39] Goudet J (1995). FSTAT (Version 1.2): a computer program to calculate F-statistics. J Heredity.

[CR40] Grant PR, Grant R, Deutsch JC (1996). Speciation and hybridization in island birds [and discussion]. Philos Trans Ser B.

[CR41] Greimler J, López P, Stuessy TF, Dirnböck T (2002). The vegetation of Robinson Crusoe Island (Isla Masatierra), Juan Fernández Archipelago, Chile. Pac Sci.

[CR42] Greimler J, López-Sepúlveda P, Reiter K, Baeza C, Peñailillo P, Ruiz E, Novoa P, Gatica A, Stuessy T (2013). Vegetation of Alejandro Selkirk Island (Isla Masafuera), Juan Fernández Archipelago, Chile. Pac Sci.

[CR43] Haberle SG (2003). Late Quaternary vegetation dynamics and human impact on Alexander Selkirk Island, Chile. J Biogeogr.

[CR44] Hardy OJ, Maggia L, Bandou E, Breyne P, Caron H, Chevallier M-H, Doligez A, Dutech C, Kremer A, Latouche-Hallé C, Troispoux V, Veron V, Degen B (2006). Fine-scale genetic structure and gene dispersal inferences in 10 Neotropical tree species. Mol Ecol.

[CR45] Hubisz MJ, Falush D, Stephens M, Pritchard JK (2009). Inferring weak population structure with the assistance of sample group information. Mol Ecol Resour.

[CR46] Hurlbert SH (1971). The nonconcept of species diversity: a critique and alternative parameters. Ecology.

[CR47] Huson DH, Bryant D (2006). Application of phylogenetic networks in evolutionary studies. Mol Biol Evol.

[CR48] Jara-Arancio P, Carmona MR, Correa C, Squeo FA, Arancio G (2012). Leaf morphological and genetic divergence in populations of *Drimys* (Winteraceae) in Chile. Genet Mol Res.

[CR49] Johnson KP, Adler FR, Cherry JL (2000). Genetic and phylogenetic consequences of island biogeography. Evolution.

[CR50] Johow F (1896) Estudios sobre la flora de las Islas de Juan Fernández. Gobierno de Chile, Santiago

[CR51] Jorgensen TH, Olesen JM (2001). Adaptive radiation of island plants: evidence from *Aeonium* (Crassulaceae) of the Canary Islands. Perspect Plant Ecol Evol Syst.

[CR52] Juan C, Ibrahim KM, Oromi P, Hewitt GM (2000). Colonization and diversification: towards a phylogeographic synthesis for the Canary Islands. Trends Ecol Evol.

[CR53] Knope ML, Morden CW, Funk VA, Fukami T (2012). Area and the rapid radiation of Hawaiian *Bidens* (Asteraceae). J Biogeogr.

[CR54] Langella O (1999) Population 1.2.30. Available from http://bioinformatics.org/project/. Accessed 01 Oct 2012

[CR55] López-Sepúlveda P, Takayama K, Greimler J, Peñailillo P, Crawford DJ, Baeza M, Ruiz E, Kohl G, Tremetsberger K, Gatica A, Letelier L, Novoa P, Novak J, Stuessy TF (2013). Genetic variation (AFLPs and nuclear microsatellites) in two anagenetically derived endemic species of *Myrceugenia* (Myrtaceae) on the Juan Fernández Islands, Chile. Am J Bot.

[CR56] Marquínez X, Lohmann LG, Faria Salatino ML, Salatino A, González F (2009). Generic relationships and dating of lineages in Winteraceae based on nuclear (ITS) and plastid (*rp*S16 and *psb*A-*trn*H) sequence data. Mol Phylogen Evol.

[CR57] Marticorena C, Quezada M (1985). Catálogo de la flora vascular de Chile. Gayana Bot.

[CR58] Marticorena C, Stuessy TF, Baeza CM (1998). Catalogue of the vascular flora of the Robinson Crusoe or Juan Fernandez Islands, Chile. Gayana Bot.

[CR59] Moore MJ, Francisco-Ortega J, Santos-Guerra A, Jansen RK (2002). Chloroplast DNA evidence for the roles of island colonization and extinction in *Tolpis* (Asteraceae; Lactuceae). Am J Bot.

[CR60] Mort ME, Soltis DE, Soltis PS, Francisco-Ortega F, Santos-Guerra A (2002). Phylogenetics and evolution of the Macaronesian clade of Crassulaceae inferred from nuclear and chloroplast sequence data. Syst Bot.

[CR61] Muñoz M **(**1980) Flora del Parque Nacional Puyehue. Editorial Universitaria, Santiago

[CR62] Muñoz-Concha D, Vogel H, Razmilic I (2004). Variación de compuestos químicos en hojas de poblaciones de *Drimys* spp. (Magnoliophyta: Winteraceae) en Chile. Revista Chilena Hist Nat.

[CR63] Nei M, Maruyama T, Chakraborty R (1975). The bottleneck effect and genetic variability in populations. Evolution.

[CR64] Nei M, Tajima F, Tateno Y (1983). Accuracy of estimated phylogenetic trees from molecular data II. Gene frequency data. J Mol Evol.

[CR65] Peakall R, Smouse PE (2006). GENALEX 6: genetic analysis in Excel. Population genetic software for teaching and research. Mol Ecol Notes.

[CR66] Percy DM, Garver AM, Wagner WL, James HF, Cunningham CW, Miller SE, Fleischer RC (2008). Progressive island colonization and ancient origin of Hawaiian *Metrosideros* (Myrtaceae). Proc Roy Soc Biol Sci Ser B.

[CR67] Pfosser M, Jakubowsky G, Schlüter PM, Fer T, Kato H, Stuessy TF, Sun B-Y (2006). Evolution of *Dystaenia takesimana* (Apiaceae), endemic to Ullung Island, Korea. Plant Syst Evol.

[CR68] Price JP, Wagner WL (2004). Speciation in Hawaiian angiosperm lineages: cause, consequence, and mode. Evolution.

[CR69] Pritchard JK, Stephens M, Donnelly P (2000). Inference of population structure using multilocus genotype data. Genetics.

[CR70] Raven PH, Kyhos DW (1965). New evidence concerning the original basic chromosome numbers of angiosperms. Evolution.

[CR71] Raymond M, Rousset F (1995). GENEPOP (version 1.2): population genetics software for exact tests and ecumenicism. J Heredity.

[CR72] Reiche C (1895). *Drimys* Forst. Anales Univ Chile.

[CR73] Ricci M (1992) Programa de conservación y recuperación de plantas amenazadas de Juan Fernández. Informe final tercera etapa. Corporación Nacional Forestal, Valparaíso

[CR74] Rodríguez RA, Quezada M (1991). Nueva combinación en *Drimys* J.R. et G. Forster (Winteraceae) de Chile. Gayana Bot.

[CR75] Rodríguez R, Quezada M (2001) Winteraceae Lindl. In: Marticorena C, Rodríguez R (eds) Flora de Chile. Universidad de Concepción, Concepción, pp 2–7

[CR76] Rodríguez R, Matthei O, Quezada M (1983) Flora arbórea de Chile. Editorial Universitaria, Concepción

[CR77] Rosindell J, Phillimore AB (2011). A unified model of island biogeography sheds light on the zone of radiation. Ecol Lett.

[CR78] Rousset F (1997). Genetic differentiation and estimation of gene flow from *F*-statistics under isolation by distance. Genetics.

[CR79] Ruiz E, Fuentes G, Becerra J, González F, Silva M (2002). Flavonoids as chemosystematic markers in Chilean species of *Drimys* J.R.Forst. et G.Forst. (Winteraceae). Bol Soc Chil Quím.

[CR80] Ruiz E, Toro O, Crawford DJ, Stuessy TF, Negritto MA, Baeza C, Becerra J (2008). Phylogenetic relationships among Chilean species of *Drimys* (Winteraceae) based on ITS sequences and insertion/deletion events. Gayana Bot.

[CR81] Schaefer H, Moura M, Belo Maciel MG, Silva L, Rumsey FJ, Carine MA (2011). The Linnean shortfall in oceanic island biogeography: a case study in the Azores. J Biogeogr.

[CR82] Schilling EE, Panero JL, Eliasson UH (1994). Evidence from chloroplast DNA restriction site analysis on the relationships of *Scalesia* (Asteraceae; Heliantheae). Am J Bot.

[CR83] Schluter D (2000). The ecology of adaptive radiation.

[CR84] Schlüter PM, Harris SA (2006). Analysis of multilocus fingerprinting data sets containing missing data. Mol Ecol Notes.

[CR85] Schönswetter P, Tribsch A (2005). Vicariance and dispersal in the alpine perennial *Bupleurum stellatum* L. (Apiaceae). Taxon.

[CR86] Simpson GG (1953). The major features of evolution.

[CR87] Skottsberg C, Skottsberg C (1921). The phanerogams of Juan Fernandez Islands. The natural history of Juan Fernandez and Easter Island, botany.

[CR88] Smith AC (1943). The American species of *Drimys*. J Arnold Arbor.

[CR89] Stuessy TF, Davis SD, Heywood VH, Hamilton AC (1995). Juan Fernández Islands. Centres of plant diversity: a guide and strategy of their conservation.

[CR90] Stuessy TF, Ebach MC, Tangney RS (2007). Evolution on specific and genetic diversity during ontogeny of island floras: the importance of understanding process for interpreting island biogeographic patterns. Biogeography in a changing world.

[CR91] Stuessy TF, Ono M, Stuessy TF, Ono M (1998). The current status of our knowledge and suggested research protocols in island archipelagos. Evolution and speciation of island plants.

[CR92] Stuessy TF, Foland KA, Sutter JF, Sanders RW, Silva M (1984). Botanical and geological significance of potassium-argon dates from the Juan Fernández Islands. Science.

[CR93] Stuessy TF, Crawford DJ, Marticorena C, Rodríguez R, Stuessy TF, Ono M (1998). Island biogeography of angiosperms of the Juan Fernandez archipelago. Evolution and speciation of island plants.

[CR94] Stuessy TF, Jakubowsky G, Salguero Gómez R, Pfosser M, Schlüter PM, Fer T, Sun B-Y, Kato H (2006). Anagenetic evolution in island plants. J Biogeogr.

[CR95] Stuessy TF, Takayama K, López-Sepúlveda P, Crawford DJ (2013) Interpretation of patterns of genetic variation in endemic plant species of oceanic islands. Bot J Linn Soc 174:276–288 (Published online 24 sep). doi:10.1111/boj.12088 10.1111/boj.12088 PMC4459035 26074627

[CR96] Sun BY, Stuessy TF, Crawford DJ (1990). Chromosome counts from the flora of the Juan Fernandez Islands, Chile. Pac Sci.

[CR97] Takayama K, López P, König C, Kohl G, Novak J, Stuessy TF (2011). A simple and cost-effective approach for microsatellite isolation in non-model plant species using small-scale 454 pyrosequencing. Taxon.

[CR98] Takayama K, Sun B-Y, Stuessy TF (2012). Genetic consequences of anagenetic speciation in *Acer okamotoanum* (Sapindaceae) on Ullung Island, Korea. Ann Bot (Oxford).

[CR99] Takayama K, Sun B-Y, Stuessy TF (2012). Anagenetic speciation in Ullung Island, Korea; genetic diversity and structure in the island endemic species, *Acer takesimense* (Sapindaceae). J Plant Res.

[CR100] Tremetsberger K, Stuessy TF, Guo Y-P, Baeza CM, Weiss H, Samuel RM (2003). Amplified fragment length polymorphism (AFLP) variation within and among populations of *Hypochaeris acaulis* (Asteraceae) of Andean southern South America. Taxon.

[CR101] van Oosterhout C, Hutchinson WF, Wills DPM, Shipley P (2004). MICRO-CHECKER: software for identifying and correcting genotyping errors in microsatellite data. Mol Ecol Notes.

[CR102] Vargas R, Cuevas JG, Le Quesne C, Reif A, Bannister J (2010). Spatial distribution and regeneration strategies of the main forest species on Robinson Crusoe Island. Revista Chilena Hist Nat.

[CR103] Vos P, Hogers R, Bleeker M, Reijans M, van de Lee T, Hornes M, Frijters A, Pot J, Peleman J, Kuiper M, Zabeau M (1995). AFLP: a new technique for DNA fingerprinting. Nucl Acids Res.

[CR104] Wallace LE, Weller SG, Wagner WL, Sakai AK, Nepokroeff M (2009). Phylogeographic patterns and demographic history of *Schiedea globosa* (Caryophyllaceae) on the Hawaiian Islands. Am J Bot.

[CR105] Weir BS, Cockerham CC (1984). Estimating F-statistics for the analysis of population structure. Evolution.

[CR106] Weller MW (1980). The island waterfowl.

[CR107] Wooten JA, Tolley-Jordan LR (2009). Validation of phylogenetic signals in amplified fragment length data: testing the utility and reliability in closely related taxa. BMC Res Notes.

[CR108] Yamada T, Maki M (2012). Impact of geographical isolation on genetic differentiation in insular and mainland populations of *Weigela coraeensis* (Caprifoliaceae) on Honshu and the Izu Islands. J Biogeogr.

