# Supplementary material for: Progressive migration and anagenesis in Drimys confertifolia of the Juan Fernández Archipelago, Chile
Source: J Plant Res. 2014 Oct 8;128(1):73–90. doi: 10.1007/s10265-014-0666-7 (PMC4300435; doi:10.1007/s10265-014-0666-7)
Supplement: Supplementary file 1 — Supplementary material 1 (DOC 154 kb) [file 10265_2014_666_MOESM1_ESM.doc]

**Table S1 *F*_ST_ values for pairwise comparisons of *Drimys confertifolia* populations. Above diagonal are estimates from AFLP data, below diagonal microsatellite data. Significance after Bonferroni correction (P < 0.05/465) is shown by asterisks.**

|  | **1** | **2** | **3** | **4** | **5** | **6** | **7** | **8** | **9** | **10** | **11** | **12** | **13** | **14** | **15** | **16** | **17** | **18** | **19** | **20** | **21** | **22** | **23** | **24** | **25** | **26** | **27** | **28** | **29** | **30** | **31** |
| --- | --- | --- | --- | --- | --- | --- | --- | --- | --- | --- | --- | --- | --- | --- | --- | --- | --- | --- | --- | --- | --- | --- | --- | --- | --- | --- | --- | --- | --- | --- | --- |
| **1** |  | -0.005 | -0.003 | 0.013 | 0.091* | 0.025 | 0.018 | 0.117 | 0.032 | 0.088* | 0.060* | 0.089* | 0.061* | 0.090* | 0.052* | 0.114* | 0.251* | 0.215* | 0.095* | 0.164 | 0.226 | 0.140* | 0.225* | 0.224* | 0.221 | 0.217 | 0.182 | 0.228* | 0.229* | 0.235* | 0.240* |
| **2** | 0.071 |  | -0.007 | 0.004 | 0.098* | 0.011 | 0.014 | 0.103* | 0.027 | 0.086* | 0.050* | 0.086* | 0.051* | 0.089* | 0.038 | 0.110* | 0.257* | 0.226* | 0.118* | 0.191 | 0.243 | 0.150* | 0.237* | 0.236* | 0.237 | 0.230 | 0.200 | 0.250* | 0.253* | 0.237* | 0.236* |
| **3** | 0.090* | 0.061 |  | -0.003 | 0.099* | 0.021 | 0.016 | 0.126 | 0.031 | 0.095* | 0.046* | 0.088* | 0.049 | 0.099* | 0.049 | 0.115* | 0.260* | 0.222* | 0.107* | 0.177 | 0.223 | 0.152* | 0.239* | 0.221* | 0.232 | 0.217 | 0.200 | 0.245* | 0.250* | 0.240* | 0.249* |
| **4** | 0.039 | 0.003 | 0.031 |  | 0.102* | 0.017 | 0.026 | 0.101 | 0.050 | 0.0810* | 0.061* | 0.087* | 0.067* | 0.090* | 0.065* | 0.113* | 0.242* | 0.211* | 0.064* | 0.167 | 0.217 | 0.120* | 0.217* | 0.204* | 0.218 | 0.208 | 0.174 | 0.229* | 0.236* | 0.227* | 0.240* |
| **5** | 0.023 | 0.053 | 0.039 | 0.022 |  | 0.075* | 0.060 | 0.046 | 0.089* | 0.021 | 0.114* | 0.027 | 0.122* | 0.026 | 0.105* | 0.024 | 0.237* | 0.208* | 0.145* | 0.192 | 0.240 | 0.055* | 0.214* | 0.278* | 0.237 | 0.216 | 0.190 | 0.241* | 0.253* | 0.217* | 0.182* |
| **6** | 0.041 | 0.033 | 0.029 | 0.019 | 0.005 |  | 0.008 | 0.070 | 0.051 | 0.058* | 0.059* | 0.053 | 0.075* | 0.068* | 0.054 | 0.085* | 0.229* | 0.2108* | 0.095* | 0.144 | 0.220 | 0.106* | 0.220* | 0.197* | 0.221 | 0.200 | 0.157 | 0.230* | 0.233* | 0.216* | 0.213* |
| **7** | 0.055 | 0.012 | 0.025 | -0.003 | 0.009 | -0.004 |  | 0.057 | 0.048 | 0.059* | 0.062* | 0.049 | 0.072* | 0.060 | 0.057* | 0.075* | 0.229* | 0.199* | 0.056* | 0.142 | 0.217* | 0.067* | 0.213* | 0.186* | 0.216 | 0.187 | 0.134 | 0.221* | 0.225* | 0.222* | 0.209* |
| **8** | 0.023 | 0.075 | 0.070 | 0.011 | 0.024 | 0.031 | 0.060 |  | 0.107 | 0.076 | 0.137* | 0.081 | 0.134 | 0.093 | 0.140* | 0.085 | 0.2306 | 0.202 | 0.128* | 0.192 | 0.288 | 0.002* | 0.217 | 0.297* | 0.277 | 0.208 | 0.135 | 0.264 | 0.273 | 0.212 | 0.160 |
| **9** | 0.157* | 0.128* | 0.144* | 0.103 | 0.132* | 0.126* | 0.151* | 0.073 |  | 0.080 | 0.007 | 0.101 | 0.014 | 0.083* | 0.011 | 0.088* | 0.283* | 0.248* | 0.161* | 0.223 | 0.273 | 0.158* | 0.260* | 0.305* | 0.279 | 0.261 | 0.223 | 0.284* | 0.281* | 0.253* | 0.237* |
| **10** | 0.027 | 0.015 | 0.059 | 0.001 | 0.021 | 0.018 | 0.010 | 0.001 | 0.099 |  | 0.102* | 0.0107 | 0.104* | 0.009 | 0.101* | 0.021 | 0.230* | 0.195* | 0.149* | 0.168 | 0.222 | 0.057* | 0.220* | 0.263* | 0.229 | 0.221 | 0.183 | 0.243 | 0.242* | 0.211* | 0.203* |
| **11** | 0.147* | 0.128* | 0.143* | 0.103* | 0.129* | 0.116* | 0.144* | 0.047 | -0.015 | 0.097* |  | 0.120* | 0.017 | 0.102* | 0.021 | 0.116* | 0.284* | 0.259* | 0.174* | 0.211 | 0.271 | 0.162* | 0.274* | 0.274* | 0.276 | 0.262 | 0.222 | 0.283* | 0.289* | 0.262* | 0.256* |
| **12** | 0.031 | 0.092 | 0.030 | 0.020 | 0.035 | 0.057 | 0.062 | 0.016 | 0.139* | 0.054 | 0.134* |  | 0.1200* | 0.013 | 0.103* | 0.026 | 0.233* | 0.194* | 0.168* | 0.172 | 0.235 | 0.106* | 0.227* | 0.263* | 0.255 | 0.225 | 0.190 | 0.238* | 0.247* | 0.227* | 0.216* |
| **13** | 0.087 | 0.026 | 0.065 | 0.017 | 0.070* | 0.042 | 0.051 | 0.063 | 0.061 | 0.035 | 0.076 | 0.084 |  | 0.107* | 0.019 | 0.121* | 0.290* | 0.254* | 0.199* | 0.217 | 0.270 | 0.174* | 0.273* | 0.293* | 0.282 | 0.265 | 0.235 | 0.291* | 0.292* | 0.267* | 0.261* |
| **14** | 0.023 | 0.018 | 0.033 | 0.001 | 0.019 | 0.028 | 0.013 | 0.037 | 0.120* | 0.025 | 0.128* | 0.022 | 0.025 |  | 0.103* | 0.006 | 0.251* | 0.220* | 0.191* | 0.203 | 0.256 | 0.103* | 0.235* | 0.302* | 0.256 | 0.248 | 0.221 | 0.259* | 0.270* | 0.242* | 0.226 |
| **15** | 0.102* | 0.059 | 0.112* | 0.023 | 0.092* | 0.092* | 0.073 | 0.044 | 0.134* | 0.044 | 0.135* | 0.104* | 0.072* | 0.058 |  | 0.104* | 0.294* | 0.266* | 0.209* | 0.234 | 0.286 | 0.217* | 0.280* | 0.297* | 0.296 | 0.265 | 0.241 | 0.293* | 0.298* | 0.270* | 0.272* |
| **16** | 0.037 | 0.054 | 0.062 | 0.027 | 0.054 | 0.067 | 0.041 | 0.025 | 0.145* | 0.020 | 0.140* | 0.045 | 0.082* | 0.029 | 0.082* |  | 0.275* | 0.239* | 0.236* | 0.226 | 0.287 | 0.135* | 0.261* | 0.342* | 0.289 | 0.270 | 0.255 | 0.283* | 0.289* | 0.256* | 0.231* |
| **17** | 0.183* | 0.181* | 0.217* | 0.152* | 0.181* | 0.179* | 0.171* | 0.171 | 0.278* | 0.129* | 0.259* | 0.235* | 0.192* | 0.156* | 0.151* | 0.179* |  | 0.019 | 0.042* | 0.076 | 0.128 | 0.153* | 0.019 | 0.167* | 0.051 | -0.026 | 0.002 | 0.057 | 0.083 | 0.094* | 0.160* |
| **18** | 0.197* | 0.201* | 0.231* | 0.178* | 0.204* | 0.199* | 0.190* | 0.200 | 0.290* | 0.155* | 0.268* | 0.249* | 0.210* | 0.167* | 0.194* | 0.185* | -0.012 |  | -0.097* | 0.002 | 0.033 | 0.054* | 0.038 | 0.001* | 0.023 | -0.001 | -0.017 | 0.024 | 0.035 | 0.098* | 0.126* |
| **19** | 0.123* | 0.129* | 0.115* | 0.119* | 0.162* | 0.143* | 0.115* | 0.179* | 0.287* | 0.134* | 0.290* | 0.120* | 0.130* | 0.039* | 0.177* | 0.111* | 0.188* | 0.170* |  | -0.108 | 0.059 | 1.000* | -0.062 | 1.000* | 0.068 | -0.071 | -0.078 | -0.053 | -0.031 | 0.069 | 0.098 |
| **20** | 0.225 | 0.228 | 0.271 | 0.195 | 0.220 | 0.224 | 0.207 | 0.240 | 0.371 | 0.154 | 0.343 | 0.280 | 0.253 | 0.198 | 0.214 | 0.210 | 0.006 | 0.049 | 0.390 |  | 0.081 | 0.027* | 0.039 | -0.051 | 0.059 | 0.047 | 0.037 | 0.0246 | 0.010 | 0.142 | 0.182 |
| **21** | 0.1450 | 0.182 | 0.170 | 0.128 | 0.151 | 0.147 | 0.161 | 0.090 | 0.293 | 0.109 | 0.266 | 0.180 | 0.203 | 0.133 | 0.160 | 0.142 | 0.005 | 0.025 | 0.232 | 0.028 |  | 0.170* | 0.071 | 0.048* | -0.024 | 0.059 | 0.141 | 0.010 | 0.013 | 0.160 | 0.229 |
| **22** | 0.1502* | 0.157* | 0.208* | 0.111* | 0.159* | 0.136* | 0.172* | 0.147* | 0.264* | 0.108* | 0.243* | 0.200* | 0.155* | 0.134* | 0.126* | 0.184* | 0.007* | 0.108* | 0.273* | 0.119* | 0.047* |  | 0.097 | 0.999* | 0.230 | 0.100 | -0.022 | 0.145 | 0.143 | 0.125 | -0.029 |
| **23** | 0.238* | 0.239* | 0.248* | 0.214* | 0.228* | 0.250* | 0.230* | 0.238 | 0.354* | 0.183* | 0.338* | 0.264* | 0.255* | 0.184* | 0.224* | 0.202* | 0.048 | 0.047 | 0.212 | 0.068 | 0.026 | 0.208 |  | 0.133* | 0.033 | -0.005 | 0.021 | 0.046 | 0.057 | 0.098* | 0.119 |
| **24** | 0.071* | 0.146* | 0.101* | 0.100* | 0.066* | 0.040* | 0.075* | 0.028* | 0.281* | 0.066* | 0.258* | 0.151* | 0.195* | 0.093* | 0.146* | 0.085* | 0.004* | 0.016* | 0.182* | 0.221* | -0.130 | 0.176* | 0.140* |  | 0.073 | 0.109 | 0.074 | -0.061 | -0.056 | 0.189 | 0.336 |
| **25** | 0.156 | 0.169 | 0.198 | 0.136 | 0.145 | 0.162 | 0.142 | 0.149 | 0.311 | 0.096 | 0.294 | 0.198 | 0.197 | 0.124 | 0.175 | 0.124 | 0.042 | 0.065 | 0.273 | -0.060 | -0.001 | 0.131 | 0.048 | 0.094 |  | 0.037 | 0.088 | -0.001 | 0.000 | 0.128 | 0.239 |
| **26** | 0.180 | 0.177 | 0.195 | 0.154 | 0.187 | 0.174 | 0.177 | 0.139 | 0.297 | 0.118 | 0.271 | 0.213 | 0.187 | 0.143 | 0.162 | 0.177 | -0.005 | 0.028 | 0.219 | 0.052 | 0.023* | 0.197 | 0.008 | 0.132 | 0.076 |  | 0.016 | 0.020 | 0.050 | 0.117 | 0.176 |
| **27** | 0.097 | 0.080 | 0.113 | 0.043 | 0.089 | 0.077 | 0.042 | 0.035 | 0.215 | 0.049 | 0.199 | 0.121 | 0.111 | 0.044 | 0.057 | 0.081 | 0.051 | 0.087 | 0.131 | 0.191 | 0.070 | 0.214 | 0.137 | -0.043 | 0.094 | 0.011 |  | 0.036 | 0.085 | 0.063 | 0.092 |
| **28** | 0.107 | 0.132* | 0.170* | 0.116 | 0.145* | 0.135 | 0.129 | 0.131 | 0.259 | 0.089 | 0.24* | 0.173* | 0.154* | 0.113* | 0.142* | 0.134* | 0.049 | 0.085 | 0.117 | 0.103 | 0.076 | 0.077 | 0.137 | 0.008 | 0.094 | 0.044 | 0.023 |  | -0.014 | 0.146* | 0.203* |
| **29** | 0.156* | 0.172* | 0.205* | 0.156* | 0.171* | 0.164* | 0.154* | 0.183 | 0.272* | 0.133* | 0.263* | 0.222* | 0.186* | 0.134* | 0.162* | 0.158* | 0.019 | 0.039 | 0.142 | 0.080 | 0.054 | 0.023 | 0.105 | -0.063 | 0.071 | 0.095 | 0.034 | 0.025 |  | 0.153* | 0.216* |
| **30** | 0.197* | 0.217* | 0.240* | 0.186* | 0.209* | 0.210* | 0.195* | 0.195 | 0.314* | 0.153* | 0.301* | 0.256* | 0.229* | 0.174* | 0.171* | 0.198* | 0.004 | 0.039 | 0.204 | 0.050 | 0.049 | 0.148 | 0.059 | 0.038 | 0.060 | -0.004 | 0.027 | 0.060 | 0.042 |  | 0.113* |
| **31** | 0.194* | 0.207* | 0.228* | 0.166* | 0.211* | 0.201* | 0.188* | 0.223 | 0.314* | 0.168* | 0.293* | 0.243* | 0.216* | 0.158* | 0.173* | 0.185* | 0.009 | 0.035 | 0.230 | 0.078 | 0.046 | 0.044 | 0.128 | 0.047 | 0.113 | 0.091 | 0.083 | 0.059 | 0.011 | 0.050 |  |
