# Supplementary material for: Progressive migration and anagenesis in Drimys confertifolia of the Juan Fernández Archipelago, Chile
Source: J Plant Res. 2014 Oct 8;128(1):73–90. doi: 10.1007/s10265-014-0666-7 (PMC4300435; doi:10.1007/s10265-014-0666-7)
Supplement: Supplementary file 2 — Supplementary material 2 (DOC 36 kb) [file 10265_2014_666_MOESM2_ESM.doc]

**Table S2** *F*_ST_ values for pairwise comparisons among populations of *Drimys winteri.* Above diagonal are estimates from AFLP data, below diagonal from microsatellite data. Significance after Bonferroni correction (P < 0.05/45) is shown by asterisks.

|  | **33** | **34** | **35** | **36** | **37** | **38** | **39** | **40** | **41** | **42** |
| --- | --- | --- | --- | --- | --- | --- | --- | --- | --- | --- |
| **33** |  | 0.285* | 0.179* | 0.255* | 0.159* | 0.279* | 0.239* | 0.176* | 0.292* | 0.231* |
| **34** | 0.093* |  | 0.216* | 0.171* | 0.223* | 0.062* | 0.134* | 0.255* | 0.208* | 0.375* |
| **35** | 0.072* | 0.091 |  | 0.188* | 0.050* | 0.209* | 0.207* | 0.199* | 0.239* | 0.238* |
| **36** | 0.183* | 0.118* | 0.128* |  | 0.192* | 0.114* | 0.154* | 0.226* | 0.200* | 0.335* |
| **37** | 0.215* | 0.167* | 0.145* | 0.122* |  | 0.218* | 0.219* | 0.183* | 0.243* | 0.214* |
| **38** | 0.160* | 0.112* | 0.159* | 0.189* | 0.172* |  | 0.130* | 0.252* | 0.200* | 0.374* |
| **39** | 0.095* | 0.105* | 0.089* | 0.110* | 0.154* | 0.162* |  | 0.147* | 0.073* | 0.264* |
| **40** | 0.166* | 0.178* | 0.166* | 0.141* | 0.179* | 0.233* | 0.043 |  | 0.194* | 0.226* |
| **41** | 0.140* | 0.189* | 0.148* | 0.163* | 0.208* | 0.241* | 0.061* | 0.035 |  | 0.251* |
| **42** | 0.223* | 0.219* | 0.226* | 0.208* | 0.236* | 0.278* | 0.088* | 0.033 | 0.130* |  |
